# Dynamic changes in radiological parameters, immune cells, selected miRNAs, and cytokine levels in peripheral blood of patients with severe COVID‑19

**DOI:** 10.3892/br.2023.1615

**Published:** 2023-03-21

**Authors:** Tetiana Bukreieva, Vitalii Kyryk, Viktoriia Nikulina, Hanna Svitina, Alyona Vega, Oleksii Chybisov, Iuliia Shablii, Oksana Mankovska, Galyna Lobyntseva, Petro Nemtinov, Inessa Skrypkina, Volodymyr Shablii

**Affiliations:** 1 Laboratory of Biosynthesis of Nucleic Acids, Department of Functional Genomics, Institute of Molecular Biology and Genetics, National Academy of Sciences of Ukraine, Kyiv 03143, Ukraine; 2 Placenta Stem Cell Laboratory, Cryobank, Institute of Cell Therapy, Kyiv 03126, Ukraine; 3 Laboratory of Cell and Tissue Cultures, Department of Cell and Tissue Technologies, State Institute of Genetic and Regenerative Medicine, National Academy of Medical Sciences of Ukraine, Kyiv 04114, Ukraine; 4 Laboratory of Pathophysiology and Immunology, D.F. Chebotarev State Institute of Gerontology of The National Academy of Medical Sciences of Ukraine, Kyiv 04114, Ukraine; 5 Department of Infectious Diseases, Shupyk National Healthcare University of Ukraine, Kyiv 04112, Ukraine; 6 Endoscopic Unit, CNE Kyiv City Clinical Hospital No. 4, Kyiv 03110, Ukraine; 7 Department of Molecular Oncogenetics, Institute of Molecular Biology and Genetics, National Academy of Sciences of Ukraine, Kyiv 03143, Ukraine

**Keywords:** COVID-19, CD4 T cells, CD8 T cells, cytokine, microRNA, immune cell subpopulations, chest CT, dynamic changes

## Abstract

The present study aimed to investigate the dynamic changes in peripheral blood leucocyte subpopulations, cytokine and miRNA levels, and changes in computed tomography (CT) scores in patients with severe coronavirus disease 2019 (COVID-19) (n=14) and age-matched non-COVID-19 volunteers (n=17), which were included as a reference control group. All data were collected on the day of patient admission (day 0) and on the 7th, 14th and 28th days of follow-up while CT of the lungs was performed on weeks 2, 8, 24 and 48. On day 0, lymphopenia and leucopenia were detected in most patients with COVID-19, as well as an increase in the percentage of banded neutrophils, B cells, and CD4^+^ Treg cells, and a decrease in the content of PD-1^low^ T cells, classical, plasmacytoid, and regulatory dendritic cells. On day 7, the percentage of T and natural killer cells decreased with a concurrent increase in B cells, but returned to the initial level after treatment discharge. The content of different T and dendritic cell subsets among CD45^+^ cells increased during two weeks and remained elevated, suggesting the activation of an adaptive immune response. The increase of PD-1-positive subpopulations of T and non-T cells and regulatory CD4 T cells in patients with COVID-19 during the observation period suggests the development of an inflammation control mechanism. The levels of interferon γ-induced protein 10 (IP-10), tumor necrosis factor-α (TNF-α) and interleukin (IL)-6 decreased on day 7, but increased again on days 14 and 28. C-reactive protein and granulocyte colony-stimulating factor (G-CSF) levels decreased gradually throughout the observation period. The relative expression levels of microRNA (miR)-21-5p, miR-221-3p, miR-27a-3p, miR-146a-5p, miR-133a-3p, and miR-126-3p were significantly higher at the beginning of hospitalization compared to non-COVID-19 volunteers. The plasma levels of all miRs, except for miR-126-3p, normalized within one week of treatment. At week 48, CT scores were most prominently correlated with the content of lymphocytes, senescent memory T cells, CD127^+^ T cells and CD57^+^ T cells, and increased concentrations of G-CSF, IP-10, and macrophage inflammatory protein-1α.

## Introduction

The disease caused by the severe acute respiratory syndrome coronavirus 2 (SARS-CoV-2) has been designated as coronavirus disease 2019 (COVID-19) by the World Health Organization. COVID-19 has spread globally, leading to a pandemic that has infected over 730 million people and caused over 6.8 million deaths (reported on February 8, 2023) in over 200 countries (https://covid19.who.int/). COVID-19 is clinically characterized by fever, fatigue, muscle pain, diarrhea, and pneumonia and can cause death in severe cases. Leukocytosis, leukopenia, and lymphopenia are commonly observed in patients with COVID-19(1). Moreover, the main feature of COVID-19 is the development of a cytokine release syndrome, which leads to acute respiratory distress syndrome (ARDS) and/or multiple-organ failure demonstrating that immunopathology plays an important role in the progression of disease severity (2-4). Exudative, proliferative, and fibrotic phases of ARDS can be triggered by a variety of clinical circumstances, including pneumonia, sepsis, and blood transfusion (5). The capillary membrane is ruptured and leaks during the first week of the exudative phase, resulting in edema, increased lung permeability, and respiratory insufficiency (6). The proliferative phase is defined by fibroblast migration through breaks in the alveolar membrane, generating a cellular granulation tissue, followed by epithelial cell withdrawal, transforming the intra-alveolar exudate into the interstitial tissue (7). The fibrotic phase, which includes substantial remodeling and collagenous tissue substitution, as well as scar formation, occurs during the third or fourth week of respiratory failure. Chest computed tomography (CT) in patients with COVID-19 is a commonly used non-invasive method for both diagnosis and management of the disease. CT is associated with disease severity and comorbidities in aged patients (8-10). It is crucial to associate clinical parameters with the formation of fibrotic lesions observed in CT in the long-term period.

Leukocytes activated within an excessive systemic inflammatory response syndrome are among the factors contributing to the pathophysiology of ARDS and inflammatory mediators. They migrate into the interstitial space of the lungs and increase endothelial permeability (11). This is accompanied by a significant influx of alveolar macrophages and neutrophils, attracted by cytokines secreted by leukocytes, followed with the destabilization of the surfactant monolayer in the air spaces, promotion of the alveolar collapse, and impairment of gas-exchange abnormalities (12).

Pro-inflammatory cytokines including tumor necrosis factor-α (TNF-α), interleukin (IL)-6, interferon γ-induced protein 10 (IP-10), monocyte chemoattractant protein-1 (MCP-1), and macrophage inflammatory protein-1α (MIP-1α) and their interactions across different cell types are other contributors to ARDS development (6). Some cells may respond to certain stimuli directly and release a specific attractant that affects a different cell type. TargeT cells respond by generating chemokines, sending out feedback signals, or recruiting a new subset of targeT cells. A cytokine storm develops as a result of this chain of events. The mechanism of cytokine release syndrome is complex and involves dysregulation of the immune cell response; therefore, strategies to control cytokine release are under investigation. Some prognostic risk factors of COVID-19 severity have already been explored, such as age, diabetes, vitamin D deficiency, IL-6 levels, N-terminal pro-B-type natriuretic peptide (NT-proBNP) levels, and serum amyloid A levels (13-17).

Circulating microRNAs (miRNAs or miRs) have already been proposed as diagnostic and prognostic markers in ARDS-related and immune pathologies. For instance, miR-27 plays an important role in reducing the inflammatory process in acute lung injury and M2 macrophage polarization (18). In addition, miR-192-5p and miR-323a-3p were reported to be differentially expressed in non-survivors and survivors of COVID-19(19). Nevertheless, the role of miRNAs in patients with COVID-19 has not been comprehensively addressed.

Numerous studies have described the clinical characteristics of patients with COVID-19, including epidemiological, clinical, laboratory, radiological, and treatment data (9,20,21). Most of these results refer to the differences between severe and non-severe patients during hospitalization or assessment of COVID-19 severity. Other reports include test results from only a single time point collected on admission, exacerbation, or discharge. However, analysis at only a single time point may conceal alterations in the parameters of an individual patient when the patient's condition changes, and it may not demonstrate diversification with disease aggravation. Recent studies analyzed a series of large sample cohorts, which included complete data on patients with COVID-19 in different disease states (1,20,22); however, there is limited research on dynamic changes in blood cell parameters and inflammatory factors to characterize disease progression and their profiling in the long-term perspective. The immune cells from the peripheral blood of a patient may be used as markers for COVID-19 and can be analyzed using fast and easily accessible blood tests (21,23,24). However, their implementation in clinical practice is limited due to the uncertainty of the mechanisms leading to changes in blood cell features and inflammatory components. The underlying fine changes in inflammatory subpopulations in peripheral blood cells, as well as changes in cytokine levels in patients with COVID-19, are ambiguous.

Therefore, in the present study, the dynamic fine changes in blood parameters, including the total number of blood cells and individual cell subpopulations, selected miRNAs, and cytokine levels in the peripheral blood of patients with severe COVID-19 were investigated over 28 days of the disease. In addition, quantitative chest CT analysis in conjunction with clinical laboratory data were used to identify prognostic factors for disease severity over a period of 48 weeks after onset of symptoms. Predictors included CT score values, blood assay parameters, and data on cytokines and miRNA levels at four time points.

## Materials and methods

### Participants

Between October and December 2020, a total of 14 confirmed cases of patients with COVID-19 (COVID-19 group) at the Kyiv City Clinical Hospital No. 4, were included in the present study. As a control group, 17 age-matched non-COVID-19 volunteer participants, who had not been hospitalized but had some underlying comorbidities, as indicated in Table I, were enrolled. The COVID-19 group included 10 male and 4 female patients between 55 and 64 years old. The volunteer group consisted of 7 male and 10 female participants aged between 36 and 67 years. The study protocol was designed in accordance with the Declaration of Helsinki and approved by the Ethics Committee of the Kyiv City Clinical Hospital No. 4 (protocol no. 280; April 23, 2020). Written informed consent was obtained from all subjects enrolled in the study. All patients met the moderate severity criteria according to the interim guidelines from the WHO and the Novel Coronavirus Pneumonia Diagnosis and Treatment Plan issued by the National Health Commission of the People's Republic of China (Provisional 7th Edition) (25). Patients from the COVID-19 group had any of the following conditions: Respiratory distress, RR ≥30 times/min; oxygen saturation (SpO2) ≤93% at rest; and bilateral pneumonia, which was observed in all enrolled participants. The major treatments for patients included drug therapy, such as antibiotic therapy/dexamethasone therapy; patients received low-pressure oxygen through a face mask while no mechanical ventilation was applied. Patients with severe COVID-19 with symptoms persisting after 7-11 days of standard treatment participated in the present study. Laboratory and clinical data from each patient were acquired for a period of 28 days, while chest CT data were acquired for 48 weeks; missing data for blood assays were due to hypercoagulation or insufficient volume.

### Respiratory pathogen detection

Laboratory validation of SARS-CoV-2 was performed at the Kyiv City Clinical Hospital No. 4 using reverse transcripton-polymerase chain reaction (RT-PCR). Briefly, throat swab specimens were obtained from the upper respiratory tract of patients and stored immediately in the viral transport medium. Following extraction of total RNA, RT-PCR was performed to identify the virus. Genotyping of the SARS-CoV-2 was not performed, but the delta strain dominated in the Ukraine at that time period.

### Blood collection

Blood samples (12-20 ml) of the 14 patients with COVID-19 were collected on the day of admission (day 0) and on days 7, 14, and 28 after admission. Briefly, 5 ml was used for routine blood assays completed using a Swelab Alfa Basic hematology analyzer (Boule Medical AB) at the Kyiv City Clinical Hospital No. 4. The remaining portions of blood samples were immediately transported to the Institute of Cell Therapy (Kyiv, Ukraine) where the plasma and serum were separated, snap-frozen, and stored at -80˚C for cytokine detection and miRNA analysis. Peripheral blood mononuclear cells (PBMCs) were isolated by density gradient centrifugation using Ficoll-Paque PLUS density gradient media (Life Sciences; Cytiva), frozen in media containing 10% DMSO (Sigma-Aldrich; Merck KGaA) and 90% fetal bovine serum (Sigma-Aldrich; Merck KGaA), and stored in liquid nitrogen until multiparametric fluorescence flow cytometry was performed.

### Flow cytometric analysis

Cryopreserved PBMCs were thawed in a water bath at 37˚C, washed with RPMI-1640 (Sigma-Aldrich; Merck KGaA) supplemented with 2% fetal bovine serum (Sigma-Aldrich; Merck KGaA), and centrifuged at 350 x g for 5 min at room temperature. Cell pellets were resuspended in RPMI-1640, filtered through a 40-µm nylon cell strainer (Corning; Corning, Inc.), and aliquoted at 50 µl into 5 ml polystyrene tubes (up to 3x10^5^ cells per tube). Cells were incubated with fluorochrome-conjugated monoclonal antibodies for 30 min at 4˚C protected from light in an appropriate dilution of 0.5 µg per 10^6^ cells. Following incubation, any unbound antibodies were washed away with 2 ml of cell wash buffer (BD Biosciences) by centrifugation at 350 x g for 5 min at 4˚C. Prior to analysis, cells were gently resuspended in 300 µl of cell wash buffer.

### Flow cytometric gating strategy

A total of seven panels of mononuclear leukocyte lineage and phenotypic markers were defined to broadly assess the immunological cellular profile of cryopreserved PBMCs: CD45/CD14/CD1c/CD11b/CD11c, CD45/CD14/CD1c/CD303/HLA-DR, CD45/CD3/CD19/CD16^+^CD56, CD3/CD4/CD8/CXCR3/HLA-DR/CD45RO, CD3/CD4/CD8/PD1/CD57/CD45RO, CD3/CD4/CD8/PD-1/HLA-DR, and CD3/CD4/CD8/CD25/CD127. To avoid inclusion in the analysis of granulocytes, CD45-positive mononuclear cells were gated out of all events by side scattering followed by subsequent singlet gating. The percentage of T cells (CD45^+^CD3^+^), B cells (CD45^+^CD19^+^), NK cells (CD45^+^CD16/56^+^), and monocytes (CD45^+^CD14^+^) were calculated among the selected mononuclear cells. Subsequent subpopulations of cells were estimated from the corresponding gated populations described above. T cells were further subdivided into Treg (CD25^+^), T memory (CD45RO^+^), T effector (CD183^+^), and activated T cells (HLA-DR^+^), as well as senescent CD57^+^ or CD279^+^ cells. Dendritic cells were further classified based on the differential expression of CD1c, CD11b, CD11c, and CD303 (Fig. S1, Fig. S2 and Fig. S3). The final relative content of each subpopulation was calculated for all CD45-positive mononuclear cells.

Antibodies used for flow cytometry and the defined lymphocyte subpopulations are listed in Table SI. To determine viable cells, 7-aminoactinomycin D dye (7-AAD; BD Biosciences) was used.

Unstained control, single stained, and fluorescence minus one controls were used to adjust the compensation settings of fluorochromes overlapping for multiparameter analysis. At least 1x10^5^-3x10^5^ cells per sample were recorded using a BD FACSAria cell sorter (Becton Dickinson; BD Biosciences). Data were analyzed using the BD FACSDiva 6.1.2 software (Becton Dickinson; BD Biosciences). The combinations of markers used to analyze distinct populations of PBMCs are listed in Table SII.

### Cytokine measurement

The C-reactive protein (CRP) content in patient sera was determined using AccuBind (cat. no. 3125-300; Monobind, Inc.) according to the manufacturer's instructions. The detection limit was 0.014 µg/ml. For the detection of granulocyte colony-stimulating factor (G-CSF), IL-2, IL-6, TNF-α, IP-10, MCP-1, and MIP-1α, enzyme-linked immunosorbent assay (ELISA) was performed using the Invitrogen kit according to the manufacturer's instructions. The following ELISA and standard curves (all from Instant ELISA; Invitrogen; Thermo Fisher Scientific) were employed for the measurement of each parameter: Human G-CSF (cat. no. BMS2001INST), IL-2 (cat. no. BMS221INST), IL-6 (cat. no. BMS213INST), TNF-α (cat. no. KHC3014), IP-10 (cat. no. BMS284INST), MCP-1 (cat. no. BMS281INST), and MIP-1α (cat. no. KAC2201). The sensitivity was 11 pg/ml for G-CSF, 2.3 pg/ml for IL-2, 0.92 pg/ml for IL-6, 0.13 pg/ml for TNF-α, 1 pg/ml for IP-10, 2.31 pg/ml for MCP-1, and 2 pg/ml for MIP-1α. All absorbance measurements were carried out using a HumaReader HS plate reader (Human GmBH). Each sample was performed in duplicate.

### miRNA expression

miRNA was extracted from the plasma of 14 patients with COVID-19 and the 17 age-matched volunteers from the control group according to the instructions for the NucleoSpin miRNA Kit (Macherey-Nagel GmbH & Company KG) and stored at -80˚C. The concentration of isolated miRNA was measured using a NanoDrop 2000 spectrophotometer (Thermo Fisher Scientific, Inc.), and miRNA was reverse transcribed into cDNA using the miRNA 1st-Strand cDNA Synthesis Kit (Agilent Technologies) with a universal reverse primer from the synthesis kit. RT-quantitative (q)PCR was conducted to detect the miRNA levels using a 5X HOT FIREPolEvaGreen qPCR Mix Plus kit (no ROX) (Solis BioDyne OÜ) with a CFX96™ Real-Time PCR Detection System (Bio-Rad Laboratories, Inc.). For each sample, the RT-qPCR reaction consisting of a 15 min hot start at 95˚C for polymerase activation, followed by 44 cycles of 15 sec at 95˚C and 20 sec at 60˚C, was performed in triplicate. The ΔΔCq method (26) was used for miRNA quantification analysis, with U6 as a reference. The primer sequences are listed in Table SIII.

### CT evaluation and scoring

Following admission, all patients lying in the supine position were subjected to high-resolution plain chest CT scanning using a Philips Brilliance CT 64 slice scanner (Philips Medical Systems Technologies, Ltd.), applying a slice thickness of 1 mm with 120 kV and 335 mAs. CT images were analyzed at weeks 2, 8, 24 and 48 after enrolment. Processing and grading of CT images considered radiologic features including ground glass opacity, reticulation, and honeycombing. The approach applied for the quantitative determination of the affected lung area was described by Büttner *et al* (27) with some changes. Briefly, the affected lung area was measured in polygonal regions of interest in one image at three levels (upper point, above the level of the carina; lower point, below the highest point of the right diaphragm; and middle point, between the previous two, right at the midpoint). Each image was parted into four quadrants with further dividing of each quadrant into 5 sub-quadrants covering 5% of the total image area. The scale applied for evaluation included 7 values: 0 (no involvement), 1 (≤10% involvement), 2 (11-20% involvement), 3 (21-30% involvement), 4 (31-40% involvement), 5 (41-50% involvement), 6 (>50% involvement). The total severity score was the sum of the scores of the five lung lobes.

### Statistical analysis

SPSS version 27.0 software (IBM Corp.) was used for statistical analysis. The variables were presented as medians with interquartile ranges. The baseline characteristics of the two groups were compared using the Chi-square test or Fisher's exact test for categorical variables or the Mann-Whitney U test for continuous variables. The Wilcoxon signed-rank test was used to compare the time-dependent events. The Mann-Whitney U test was used to compare the differences between the groups at each time point. The Spearman rank test was performed to assess the correlations between variables. GraphPad Prism software (version 7.0a; GraphPad Software, Inc.) was used for the data visualization. A P-value of ≤0.05 was considered to indicate a statistically significant difference.

## Results

### Basic characteristics of the patients with COVID-19

A total of 14 patients with severe COVID-19 admitted to the Kyiv Clinical Hospital No. 4 were enrolled in the present study after obtaining written informed consent. The detailed patient characteristics are shown in Table I. The median age of the COVID-19 and control groups were 62.0 (55-64.0) and 62.0 (36.2-67.0) years, respectively, and the interval from illness onset to hospital admission for the COVID-19 group was 11.0 (8.5-12.8) days.

### Dynamic profile of hematological parameters in patients with COVID-19

**Figure 1:**
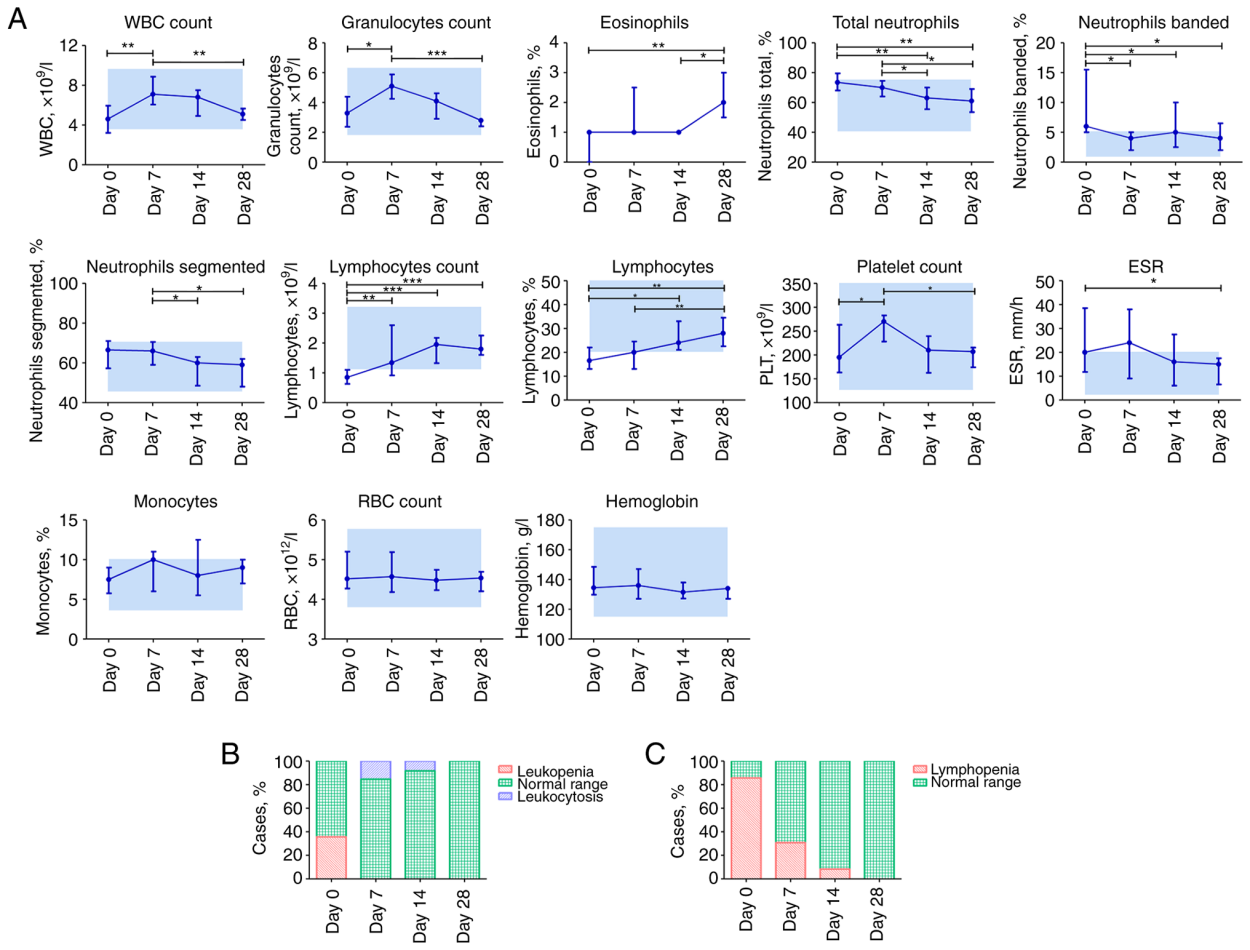
Dynamic changes in hematological parameters in patients with COVID-19. (A) Dynamic profile of blood cell types. Blue rectangles denote the normal range. (B) Ratio of leukopenia and leukocytosis. (C) Ratio of lymphopenia. Data are presented as the median and interquartile range. Wilcoxon signed-rank test: ^*^P≤0.05, ^**^P≤0.01 and ^***^P≤0.001. COVID-19, coronavirus disease 19; WBC, white blood cells; ESR, erythrocyte sedimentation rate; RBC, red blood cells.

Blood parameter comparisons in patients with COVID-19 depending on the time of assessment are presented in Fig. 1A. White blood cell (WBC) and granulocyte counts increased on day 7 and steadily decreased by day 28, whereas the percentage of neutrophils, including banded and segmented, decreased gradually from the time of admission on day 0 to 28. However, the percentages and counts of lymphocytes increased regularly. Compared to day 0, the percentage of eosinophils increased significantly on day 28 (P≤0.01). Platelet count showed a sharp increase on day 7; however, all values appeared within the normal range (125.0-350.0x10^9^/l). A significant decrease in erythrocyte sedimentation rate (ESR) was observed only on day 28 compared to the initial day (P≤0.05). Other parameters, including the percentage of monocytes, red blood cell count, and hemoglobin, were not altered during COVID-19 progression and early recovery.

On day 0, 5/14 (35.71%) patients with COVID-19 had leucopenia, whereas 2/13 (15.38%) patients had leukocytosis on day 7. Lymphopenia (≤1.1x10^9^/l) occurred in 12/14 (85.71%) patients with COVID-19 on day 0 and was not observed on day 28 (Fig. 1B).

### Dynamic profile of lymphocyte cell subpopulations in patients with COVID-19

**Figure 2:**
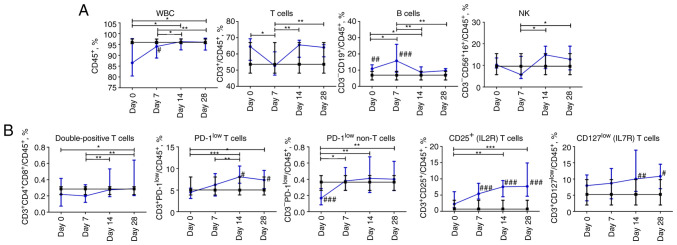
Dynamic changes in lymphocyte subsets in patients with COVID-19. (A) Frequency of white blood cells, T cells, B cells, natural killer cells. (B) Frequency of double-positive CD4^+^CD8^+^ T cells, CD3^+^PD-1^low^ T cells, CD3^-^PD-1^low^ non-T cells, CD25-expressing, and CD127-expressing T cells. Data are presented as the median and interquartile range. Wilcoxon signed-rank test was used for comparison of time-dependent events: ^*^P≤0.05, ^**^P≤0.01 and ^***^P≤0.001. Mann-Whitney U test was used for comparing the values of the control and COVID-19 groups at each time point: ^#^P≤0.05, ^##^P≤0.01 and ^###^P≤0.001. The blue line represents the COVID-19 group; and the black line represents the control group. COVID-19, coronavirus disease 19; WBC, white blood cells; NK, natural killer.

The frequencies of major lymphocyte subsets in the peripheral blood of the patients with COVID-19 are shown in Fig. 2A. The percentage of CD45^+^ WBC on day 7 was significantly lower than that in the control group (P≤0.05) but restored on day 14. The median value of T cell (CD45^+^CD3^+^) content in the COVID-19 group decreased at day 7 and returned to significantly elevated levels on days 14 and 28 (P≤0.05). The percentage of B cells (CD19^+^) peaked on day 7 compared to that on days 14-28 and differed significantly from those of the control group during the first week of hospitalization. The percentage of natural killer (NK) cells (CD3^-^CD16^+^CD56^+^) decreased from the start of observation reaching a nadir on day 7 of hospitalization and then increased constantly but not significantly through the next two weeks.

The content of double-positive (DP) T cells CD3^+^CD4^+^CD8^+^ among mononuclear leukocytes was under-represented on days 0 and 7 and restored to values of the control group on days 14 and 28. The frequencies of PD-1-expressing cells in both CD3^+^ and CD3^-^ blood cell populations increased during the four weeks of assessment. A significant increase in CD3^+^ PD-1 expressing blood cells occurred from 7 to 14 days (P≤0.01), whereas CD3^-^ PD-1 expressing cells increased during the first week of hospitalization and then reached a plateau. The percentage of PD-1^low^ T cells was significantly higher in COVID-19 patients on days 14 and 28 compared to the control group. The percentage of PD-1^low^ non-T cells was significantly lower at the beginning of hospitalization compared to the cohort of non-COVID-19 volunteers. The content of CD25^+^ T cells increased during the observation period and was significantly higher than that in the control group, from day 7 onward. The percentages of CD127^+^ T cells demonstrated consistent slight growth during the observation period, reaching significant differences compared to those of the control group on days 14 and 28 (Fig. 2B).

### Dynamic profile of changes in subpopulations of T cells in patients with COVID-19. CD8 T cells

**Figure 3:**
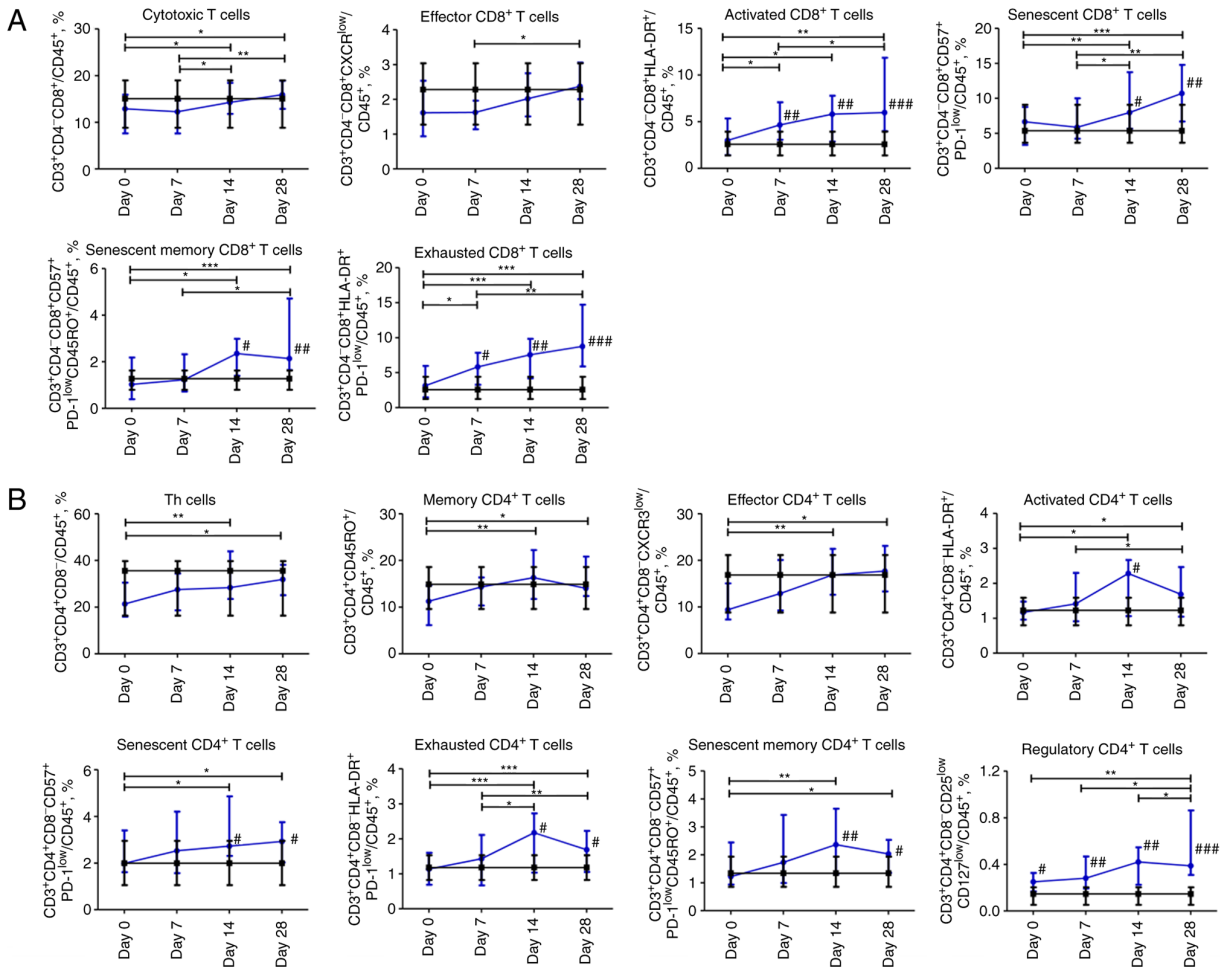
Dynamic changes in T-cell subsets in patients with COVID-19. (A) Different subsets of CD8 T cells. (B) Different subsets of CD4 T cells. Data are presented as the median and interquartile range. Wilcoxon signed-rank test was used for comparison of time-dependent events: ^*^P≤0.05, ^**^P≤0.01 and ^***^P≤0.001. Mann-Whitney U test was used for comparing the values of the control and COVID-19 groups at each time point: ^#^P≤0.05, ^##^P≤0.01 and ^###^P≤0.001. The blue line represents the COVID-19 group; and the black line represents the control group. COVID-19, coronavirus disease 19.

The content of cytotoxic CD3^+^CD4^-^CD8^+^ T cells and effector CD3^+^CD4^-^CD8^+^CXCR3^low^ T cells steadily increased from day 0 to 28. The content of activated CD3^+^CD4^-^CD8^+^HLA-DR^+^ and exhausted CD3^+^CD4^-^CD8^+^HLA-DR^+^PD-1^low^ T cells increased during the entire observation period and was significantly higher on days 7, 14, and 28 in comparison with non-COVID-19 volunteers. The percentage of senescent CD8 T cells CD3^+^CD4^-^CD8^+^CD57^+^PD-1^low^ and their memory subpopulation CD3^+^CD4^-^CD8^+^CD57^+^PD-1^low^CD45RO^+^ began to increase on day 7 and reached the maximum value on day 28. Compared to the control group, the content of senescent CD8^+^ T cells and the memory subpopulation of senescent CD8^+^ T cells was significantly higher on days 14 and 28 (P≤0.05 and P≤0.01, respectively; Fig. 3A).

### CD4 T cells

The percentage of CD3^+^CD4^+^CD8^-^Th cells increased significantly from the day 0 to 28. The memory CD4^+^ T cells CD3^+^CD4^hi^CD45RO^+^ subpopulation increased steadily throughout the four weeks. The effector CD4^+^ T cells CD3^+^CD4^+^CD8^-^CXCR3^low^ subpopulation increased over 14 days and did not change during the fourth week. The abovementioned CD4 T cell subpopulations were comparable to those of the control group. The population of activated CD4 CD3^+^CD4^+^CD8^-^HLA-DR^+^ T cells increased for 28 days, with a peak value on day 14 acquiring a significant difference (P≤0.05) compared to the control group. Exhausted CD4 CD3^+^CD4^+^CD8^-^HLA-DR^+^PD-1^low^ and senescent memory CD3^+^CD4^+^CD8^-^CD57^+^PD-1^low^CD45RO^+^ T cells shared the same pattern as activated CD4 T cells. The percentage of senescent CD4 T cells CD3^+^CD4^+^CD8^-^CD57^+^PD-1^low^ increased over 28 days. The senescent, senescent memory, and exhausted cells in the COVID-19 group were significantly abundant (P≤0.05) on days 14 and 28. The percentage of CD4 regulatory T cells CD3^+^CD4^+^CD8^-^CD25^low^CD127^low^ increased and remained significantly higher on days 0 (P≤0.05), 7 (P≤0.01), 14 (P≤0.01), and 28 (P≤0.001) compared to the control group (Fig. 3B).

### Dynamic profile of changes in subpopulations of myeloid mononuclear cells in patients with COVID-19

**Figure 4:**
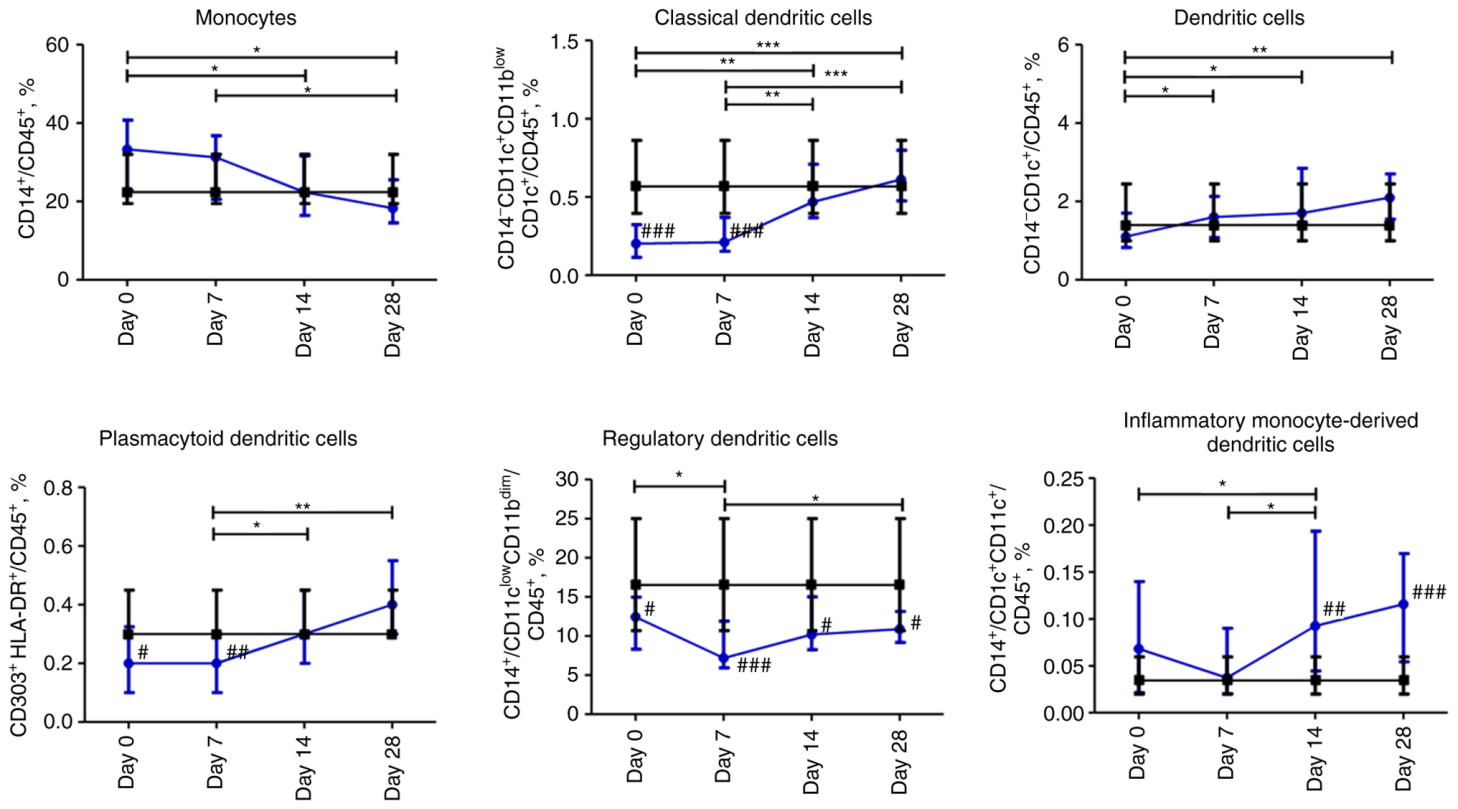
Dynamic changes in myeloid mononuclear cell subsets in patients with COVID-19. Data are presented as a median and interquartile range. Wilcoxon signed-rank test was used for comparison of time-dependent events: ^*^P≤0.05, ^**^P≤0.01 and ^***^P≤0.001. Mann-Whitney U test was used for comparing the values of the control and COVID-19 groups at each time point: ^#^P≤0.05, ^##^P≤0.01 and ^###^P≤0.001. The blue line represents the COVID-19 group; and the black line represents the control group. COVID-19, coronavirus disease 19.

The percentage of CD45^+^CD14^+^ monocytes gradually decreased over 28 days. The content of CD14^-^CD11c^+^CD11b^low^CD1c^+^ dendritic cells was significantly lower on day 0 and 7 (P≤0.001) compared to the control group; however, it normalized on days 14 and 28. The content of CD14^-^CD1c^+^ dendritic cells increased during the observation period and was within the normal range. The content of plasmacytoid dendritic cells CD303^+^HLA-DR^+^ was significantly lower over the first 7 days than in the control group, and then, it significantly increased from day 7 to 28 (P≤0.01). Compared to non-COVID-19 volunteers, the percentage of regulatory CD14^+^CD11b^dim^CD11c^low^ dendritic cells was significantly under-represented over four weeks of assessment with a nadir on day 7. The percentage of inflammatory monocyte-derived CD14^+^CD1c^+^CD11c^+^ dendritic cells gradually increased from day 7 to 28 and reached a significant difference at days 14 and 28 (P≤0.01 and P≤0.001, respectively) compared to the control group (Fig. 4).

### Cytokine and miRNA levels in the plasma of patients with COVID-19

**Figure 5:**
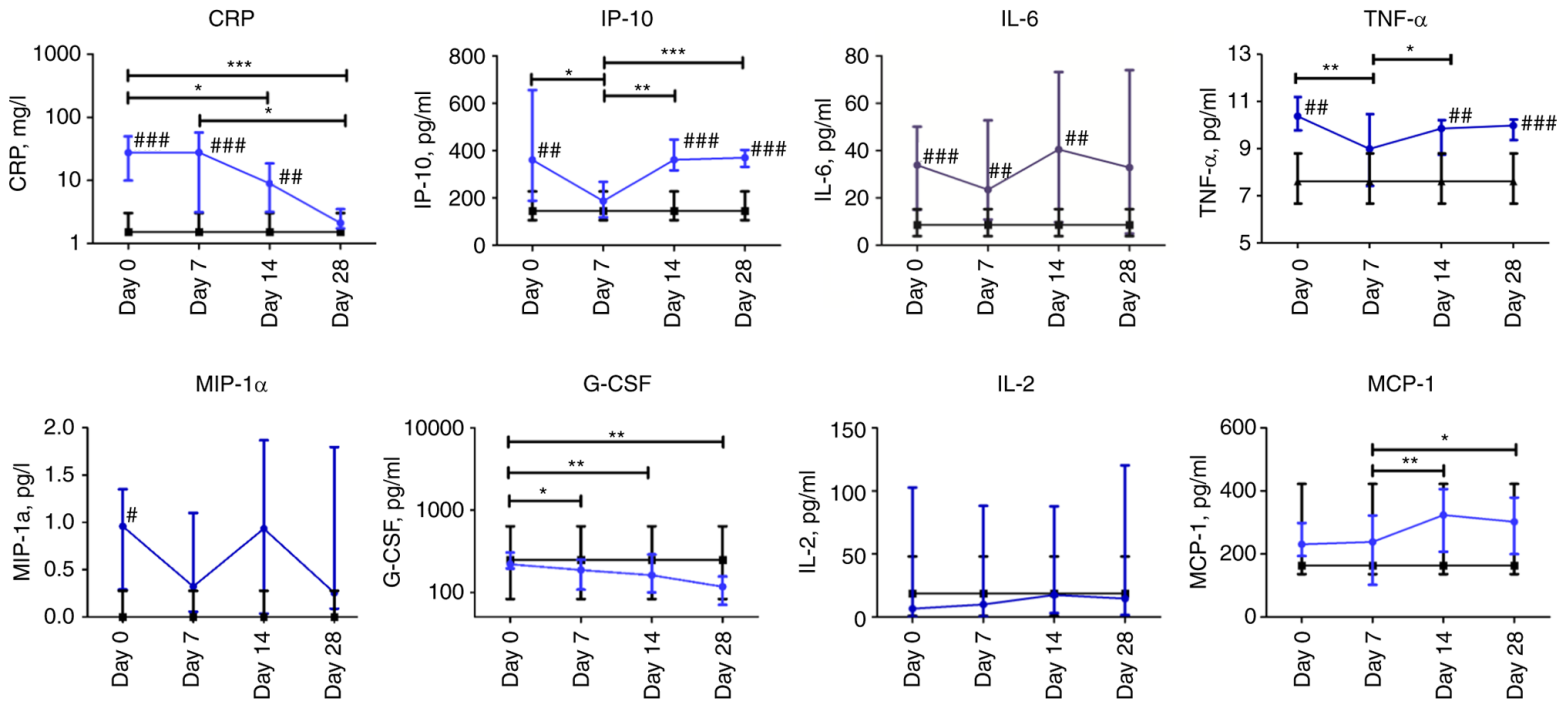
Dynamic changes in cytokine levels in patients with COVID-19. Data are presented as a median and interquartile range. Wilcoxon signed-rank test was used for comparison of time-dependent events: ^*^P≤0.05, ^**^P≤0.01 and ^***^P≤0.001. Mann-Whitney U test for comparing the control and COVID-19 groups at each time point: ^#^P≤0.05, ^##^P≤0.01 and ^###^P≤0.001. The blue line represents the COVID-19 group; and the black line represents the control group. COVID-19, coronavirus disease 19; CRP, C-reactive protein; IP-10, interferon γ-induced protein 10; IL-6, interleukin 6; TNF-α, tumor necrosis factor-α; MIP-1α, macrophage inflammatory protein-1α; G-CSF, granulocyte colony-stimulating factor; IL-2, interleukin 2; MCP-1, monocyte chemoattractant protein-1.

During two weeks of hospitalization, CRP levels in the plasma of patients with COVID-19 were significantly higher than in the plasma of volunteers in the control group and decreased from day 7 to 28 of observation. IP-10 levels in the plasma of patients with COVID-19 were increased compared to those in the control group, except for a drop to normal ranges on day 7. TNF-α levels were significantly higher on days 0, 14, and 28 in the COVID-19 group, with a slight reduction on day 7 compared to the control group. The concentration of MIP-1α was higher at the beginning of observation in COVID-19 patients in comparison with the control group. Furthermore, the levels of MIP-1α, IL-6, and IL-2 were not altered throughout the observation period in the COVID-19 group between different time points. However, IL-6 values differed significantly from the control group on days 0 (P≤0.001), 7 (P≤0.01), and 14 (P≤0.01). The concentration of G-CSF gradually decreased over the observation period in the COVID-19 group and MCP-1 levels in the plasma of patients with COVID-19 increased from day 7 (Fig. 5).

**Figure 6:**
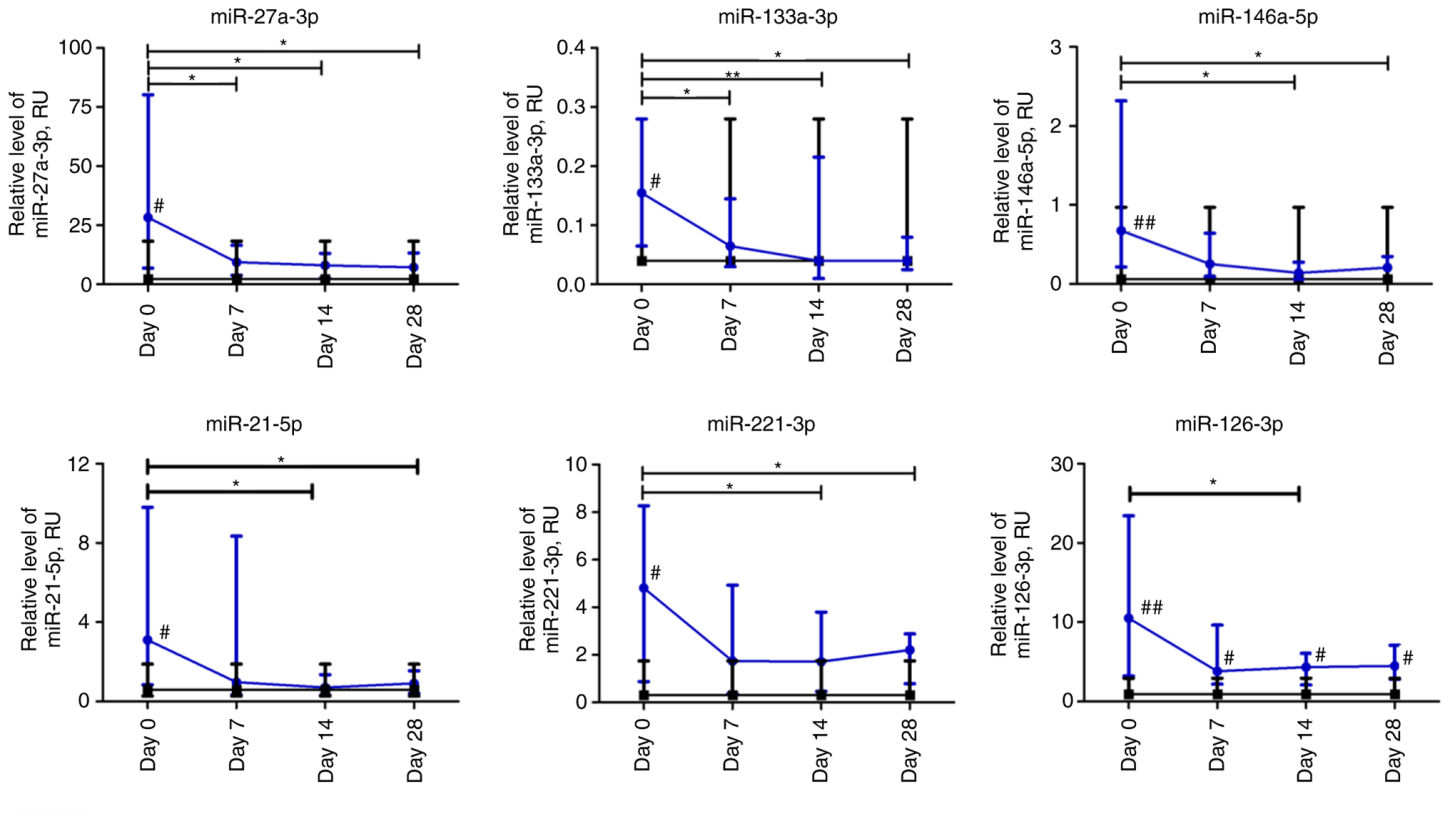
Dynamic changes in miRNA expression levels in patients with COVID-19. Data are presented as a median and interquartile range. Wilcoxon signed-rank test was used for comparison of time-dependent events: ^*^P≤0.05 and ^**^P≤0.01. Mann-Whitney U test was used for comparing the values of the control and COVID-19 groups at each time point: ^#^P≤0.05 and ^##^P≤0.01. The blue line represents the COVID-19 group; and the black line represents the control group. miRNA or miR, microRNA; COVID-19, coronavirus disease 19; RU, relative units.

At the beginning of observation, all investigated miRs had significantly higher expression levels compared to non-COVID-19 volunteers. The relative expression levels of miR-27a-3p and miR-133a-3p in patients with COVID-19 decreased significantly over the first week and remained at a low level up to day 28 compared to day 0 (P≤0.05 and P≤0.01, respectively). The relative expression levels of miR-146a-5p, miR-21-5p, and miR-221-3p in the plasma of patients with COVID-19 were significantly lower on days 14 and 28 compared to day 0. The relative level of miRNA expression was altered most markedly on day 14 compared to day 0. For example, the relative expression levels of miR-21-5p and miR-146a-5p exhibited a 4-fold decrease, whereas that of miR-221-3p exhibited a 3-fold decrease. The relative expression level of miR-126-3p in the COVID-19 group significantly differed at day 14 compared to the day 0 time point. The expression of all investigated miRNAs markedly differed from the control group on day 0 (P≤0.05). Moreover, miR-126-3p remained significantly higher in COVID-19 patients during the four weeks of observation compared to the control group (Fig. 6).

### CT of the lungs

**Figure 7:**
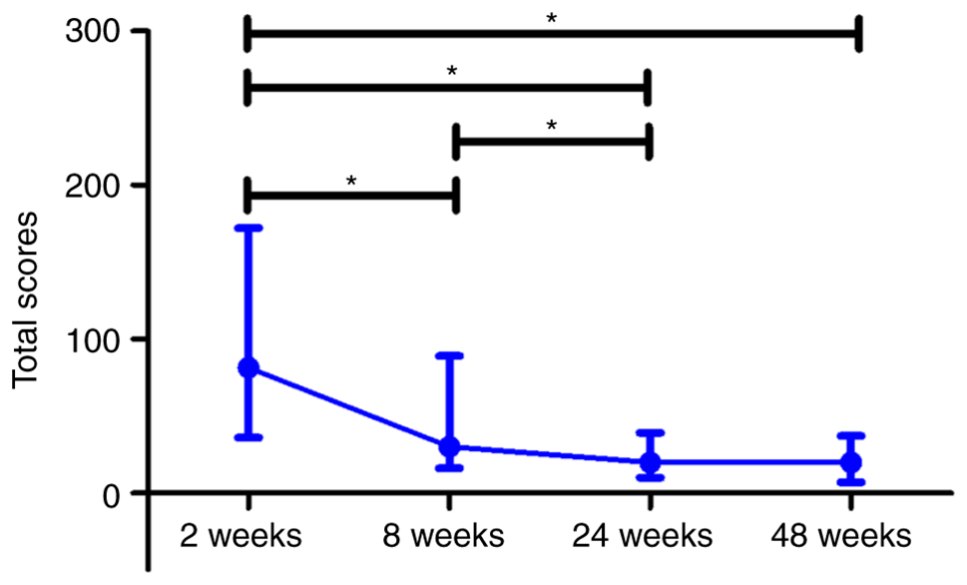
Dynamic changes in lung computed tomography scores in patients with COVID-19. Data are presented as a median and interquartile range. Paired t-test was used for comparison between related values of time-dependent events: ^*^P≤0.05. The blue line represents the COVID-19 group. COVID-19, coronavirus disease 19; wk, week.

The total lung CT score of patients with COVID-19 gradually decreased over all the observation period with significant differences on weeks 8, 24 and 48 compared to week 2 (P≤0.05). In addition, the CT score differed significantly between week 8 and 24 (P≤0.05). Concurrently, the median value for the CT total score did not significantly differ between weeks 24 and 48 after the beginning of hospitalization (Fig. 7).

### Correlation between lymphocyte subsets, cytokines, and miRNA

The most significant data obtained using correlation analysis are presented in Fig. S4. CRP levels were positively correlated with ESR (r=0.545, P≤0.0001), neutrophil percentage (r=0.688, P≤0.0001), and granulocytes (r=0.467, P≤0.0001) and negatively correlated with eosinophils (r=-0.428, P≤0.001) and lymphocyte count (r=-0.613, P≤0.0001) and percentage (r=-0.731, P≤0.0001). A negative correlation was revealed between CRP and plasmacytoid dendritic cells (r=-0,481, P≤0.001) and classical dendritic cells (=-0.589, P≤0,0001). A positive correlation was found between the concentration of G-CSF and ESR (r=0.424, P≤0.005) and neutrophil percentage (r=0.443, P≤0.001) and a negative correlation was revealed with lymphocyte count (r=-0.448, P≤0.001). Additionally, G-CSF was highly correlated with the expression of miR-27a-3p (r=0.401, P≤0.001), miR-21-5p (r=0.304, P≤0.01), miR-146a-5p (r=0.321, P≤0.01), miR-221-3p (r=0.302, P≤0.01), and miR-133a-3p (r=0.351, P≤0.005).

miR-126-3p expression was positively correlated with inflammatory monocyte-derived dendritic cells (r=0.314, P≤0.01), CD3^+^CD127^hi^ cells (r=0.336, P≤0.005) and negatively with regulatory dendritic cells (r=-0.334, P≤0.005) (data not shown). miR-146a-5p negatively correlated with WBC (r=-0.379, P≤0.005). The plasma level of miR-21-5p was negatively correlated with dendritic cells (r=-0.358, P≤0.005) and senescent memory CD8 T cells (r=-0.333, P≤0.005). A positive correlation was revealed between miR-133a-3p and IL-6 (r=0.316, P≤0.01).

The subset of regulatory dendritic cells was positively correlated with the content of NK cells (r=0.503, P≤0.0001) and negatively with B cells (r=-0.487, P≤0.0001). In addition, plasmacytoid dendritic cells were negatively correlated with neutrophils (r=-0.405, P≤0.005) and positively with classical dendritic cells (r=0.460, P≤0.0001). CD3^+^CD25^+^ was strongly correlated with the content of following CD8 T cells subsets: Activated (r=0.810, P≤0.0001), senescent (r=0.701, P≤0.0001), senescent memory (r=0.591, P≤0.0001), and exhausted CD8 T cells (r=0.854, P≤0.0001). The analysis revealed a negative correlation between CD14^+^ myeloid cells and T-cell populations as follows: DP CD4^+^CD8^+^ (r=-0.325, P≤0.001), Th cells (r=-0.807, P≤0.0001), effector Th cells (r=-0.731, P≤0.0001), cytotoxic T cells (r=-0.311, P≤0.01), memory Th cells (r=-0.624, P≤0.0001), PD-1^low^ T cells (r=-0.499, P≤0.0001), and CD127^low^ T cells (r=-0.470, P≤0.0001).

### Correlation between laboratory parameters and CT

The most significant data on the correlation analysis with CT scores are presented in Table II. The CT score was negatively correlated with lymphocyte count and positively with ESR. The CT parameters of lung injury were negatively correlated with the content of CD4^+^ among CD45^+^ cells. The percentage of senescent memory CD4^+^ and CD8^+^ T cells among CD45^+^ cells was positively correlated with CT lesions. CD3^+^CD57^hi^ and CD3^+^CD127^hi^ cells were strongly positively and negatively correlated, respectively, with the CT scores of the lung. A positive correlation was revealed between the lung lesion score and G-CSF, IP-10, and MIP-1α concentration on weeks 2, 8, 24 and 48.

## Discussion

In the present study, the dynamic changes in blood parameters, including alterations in individual cell subpopulations, selected miRNAs, and cytokine levels in the peripheral blood of patients with severe COVID-19 over 28 days of the disease and their association to lung lesions in the long term were assessed.

It was observed that WBC and granulocyte counts increased on day 7 and decreased on day 28, whereas the percentage of neutrophils, including banded and segmented, decreased gradually. However, an opposite trend in the percentage and count of lymphocytes was observed, demonstrating a regular increase. Similar dynamics in increased neutrophils versus decreased lymphocytes in patients with severe COVID-19 have been previously reported (28). Another study also showed that critical patients with COVID-19 pneumonia have an immune deficiency, which may lead to serious infection and mortality (23). The reduction in lymphocytes may be caused by the dysregulation in cytokine production (29), destruction of lymphatic organs (30), and migration of CD8^+^ circulating lymphocytes to the lungs (24,31). This data coincides with a negative correlation between lymphocyte count and lung lesions revealed in the present study.

The observed decrease in the percentage of T cells and NK cells among PBMCs on day 7 of hospitalization and concurrent increase in the percentage of B cells may be associated to corticosteroid treatment (32,33). In general, no changes in the B lymphocyte population were detected in other study groups (34,35).

The findings in the present study showed higher frequencies of PD-1-positive T cells at different time points in patients with COVID-19 compared to day 0. PD-1 downregulates the proliferation and production of cytokines by T cells and controls the damage to normal tissues during infection (36). Moreover, an increased percentage and absolute count of PD-1-expressing CD3^+^CD4^+^ and CD3^+^CD8^+^ T cells have been previously reported in autoimmune diseases (37). The formation of an inflammatory control mechanism by the increase in PD-1-positive T and non-T cell subpopulations as well as regulatory CD4 T cells in patients with COVID-19 across the whole observation period is suggested.

CD3^+^CD4^+^CD8^+^ DP T cells are a distinct, minor population of cells that are particularly detectable in viral infections and have both cytotoxic and immunosuppressive properties (38,39). The increase of DP T cells with CD3^+^CD4^+^CD8^+^ immunophenotype among mononuclear WBCs in the process of recovery from COVID-19 was revealed. This may indicate their functional significance in the fight against persistent infections. However, the absolute counts of CD3^+^CD4^+^CD8^+^ DP T lymphocytes progressively decreased in patients with more severe COVID-19(21).

A consistent increase in the content of CD25^+^ and CD127^+^ T cells over 28 days was observed. Furthermore, the content of CD127^+^ T cells was negatively correlated with lung injuries. Upregulated expression of CD25^+^ on T cells from patients with severe COVID-19 has also been reported (40,41). Moreover, Chen *et al* (42) revealed that the number and proportion of CD4^+^CD25^+^CD127^low^ cells increased in both patients with mild and severe COVID-19, compared to the control group, and remained at higher levels after recovery. Additionally, CD127-expressing T cells are considered to be SARS-CoV-2-specific long-lived T cells (43). Signaling by CD127/IL-7 is involved in numerous key aspects of T-cell survival and proliferation, therefore, increased CD127 expression levels on T cells could be involved in overcoming lymphopenia in patients with COVID-19 and thus lead to a decrease in lung inflammation. Furthermore, it was shown that an increase in the content of activated effector T cells with CD4^+^CD25^+^CD127^high^ phenotype has a significant negative correlation with multiple organ failure (44). In the present study, the content of CD3^+^CD25^+^ T cells was strongly associated with different populations of activated, senescent, and exhausted CD8^+^ T cells. Arguably, CD25-expressing activated T cells receive IL-2 signaling, which further in a positive feedback manner promotes their proliferation and differentiation (45). During the observation period, an increased content of CD4 T-cell subsets (memory, effector, activated, senescent, senescent memory, and exhausted) and CD8 T cells (effector, activated, senescent memory, and exhausted CD8 Т cells) was noted, suggesting the activation of an adaptive immune response against the inflammation progression in the lungs. In addition, abundant content of activated both CD4^+^ and CD8^+^ T cells is a characteristic of COVID-19 increasingly studied (45,46). Dysregulation of reactive CD8^+^ and CD4 T cells has been described in the early phase of immune response and immune memory development (47-49). The data in the present study are consistent with those of a previous study that reported an association between a high level of CD57 expression among CD8^+^ T cells and immune senescence with either human aging or prolonged chronic infections in patients with severe COVID-19(50). The association that was revealed between the proportions of senescent memory T cells and CD3^+^CD57^hi^ cells with the lung lesion indicates that lymphocytes are one of the key players in the pathogenesis of lungs during COVID-19.

In the present study, a strong correlation was detected between both activated CD4 and CD8 T cells and the PD-1 expression levels. Similarly, a high correlation was previously revealed between PD-1-expressing cells and activated CD38^+^HLA-DR^+^ CD4 T cells, but not with activated CD8 T cells (42). Notably, activated T cells also increase the expression of the activation markers CD38 and HLA-DR (48). From day 7, a significant increase in the percentage of Treg cells was observed. Certain studies revealed a lower level of Tregs in severe patients than in mild or moderate patients (51,52), whereas Chen *et al* indicated a trend toward higher content of Tregs in the severe group (53). Moreover, the dynamics of Treg frequency with a gradual increase from day 7 that peaked on day 22 compared to healthy donors, and a slight decrease up to day 28 was reported in a case report of an asymptomatic COVID-19 patient (54). It is considered that all these discrepancies in results occurred due to variance in the time of assessment and do not comprise the full pattern of dynamic changes in cell subpopulations during the course of COVID-19.

It was revealed that the percentage of classical and plasmacytoid dendritic cells was significantly lower on days 0 and 7 but restored from day 14 in the COVID-19 group. These data are consistent with the previously reported lower frequency of CD1c^+^ dendritic cells in peripheral blood in patients with severe COVID-19 due to their increased migration to the lungs (55). Moreover, it was reported that lower percentages of plasmacytoid and myeloid dendritic cells were observed in the blood of patients with severe COVID-19 compared to healthy donors (34).

The repeated increase in the content of CD45^+^CD11c^low^CD11b^dim^ regulatory dendritic cells on days 14-28 after a slight decrease on day 7, is considered to be an indicator of prolonged inflammation. The data in the present study, are consistent with the previously reported considerable increase in this cell population that was almost restored to normal levels after intravenous transplantation of mesenchymal stem cells in patients with severe COVID-19(56). Thus, it is surmised that regulatory dendritic cells are essential for the excessive production of proinflammatory cytokines and further aggravate infection by suppressing T-cell functions (57). Interestingly, regulatory dendritic cells were positively correlated with NK cells and negatively with B cells.

Furthermore, it was hypothesized that the detected under-represented classical dendritic cell content up to day 7, with a concurrent increase in the percentage of plasmacytoid dendritic cells observed after day 7, may be a consequence of corticosteroid therapy (58).

In the present study, a gradual increase in the percentage of inflammatory monocyte-derived dendritic cells along with regulatory T cells was observed, implying the formation of adaptive immunity. These cells have been shown to considerably increase in numbers and promote the differentiation of memory CD8^+^ T cells during acute viral infection (59). In addition, T-cell mediated-response to the new coronavirus was shown in individuals with asymptomatic or mild symptoms of COVID-19(60). Moreover, inflammatory dendritic cells are involved in innate defense and T-cell activation in a pathogen-dependent manner (61,62).

Consistent with previous studies (63-65), the present study confirmed that the CRP level is a useful biomarker in the early stages of COVID-19 infection . Based on the data of the present study, although CRP reached the normal value on day 28 in patients with COVID-19, the levels of several subpopulation of T cells remained elevated. Therefore, the levels of pro-inflammatory cytokines should be evaluated to control the general inflammation status. In the present study, similar to a previous study (66), a positive correlation between CRP and inflammatory parameters including ESR, granulocytes, and neutrophils was observed. While a negative correlation was revealed between CRP levels and classical and plasmacytoid dendritic cells that coincides with another study (67). Furthermore, it was revealed that CRP is strongly and inversely related to lymphocytes as was previously reported (68,69).

Previously, high levels of G-CSF were detected in both intensive care unit (ICU) and non-ICU patients compared to healthy volunteers (70). In the present study, it was revealed that G-CSF levels were positively correlated with neutrophils and negatively with lymphocytes. In addition, it was shown that the plasma levels of both G-CSF and MIP-1α were positively correlated with CT scores. It was reported that the progression of inflammation in patients with COVID-19 may be related to the amount of G-CSF (71). However, the correlation between G-CSF, NLR, and the prognosis is still being debated, as is the utilization of dynamic changes in G-CSF and immune cells content to predict disease course and response to therapy (72,73). It was also found that the administration of corticosteroids can significantly reduce the concentration of IP-10 in the plasma of patients with COVID-19(20). In the present study, a decrease was observed in plasma IP-10 concentration on day 7 and its return to baseline through the second week, in contrast to the levels of IL-6 and CRP. It is hypothesized that it may be associated with the discontinuation of corticosteroid therapy. Thus, the plasma levels of IP-10 and MCP-1 in critically ill patients were significantly higher than those in severe patients (74). Moreover, IL-6 and MCP-1 are considered as the main risk factors related to mortality in hospitalized COVID-19 patients (75). In the present study, the plasma concentration of IP-10 was correlated with lung lesions that coincides with the aforementioned data. However, in the present study, the levels of IL-2 and IL-6 were not altered during the observation period. Most studies reported an increase in IL-2 levels in patients with severe COVID-19 (23,76,77), whereas, in other studies, IL-2 levels remained within the normal range during the treatment period (78,79). As previously reported, the level of IL-6 was higher than in healthy donors, and similarly to the data of the present study, it was not significantly altered during the observation period (42,78). A high level of TNF-α in the serum and/or plasma of patients with COVID-19 reported in previous studies (29,56,80) is consistent with the findings of the present study indicating abnormally activated host immune cells.

It was observed that the relative expression levels of miR-21-5p, miR-221-3p, miR-27a-3p, miR-133a-63p, and miR-146a-5p were significantly higher at the beginning of hospitalization and decreased within two weeks of treatment. miR-146a has been previously reported to be an important molecular suppressor of inflammation through its capacity to target members of TLR and NF-κB signaling, as well as the proteoglycan family (81). miR-146a reduces NF-κB-dependent pro-inflammatory cytokines in TNF-α-stimulated monocytes (82). Additionally, an increase in the expression level of miR-146a was revealed to reduce lung cell damage by suppressing inflammatory responses (83). Moreover, a high expression level of miR-146a-5p in plasma in the COVID-19 patient group compared to the healthy group has been previously shown (84). Furthermore, in the present study, a negative correlation between the level of miR-146a and WBC was observed. It has been previously reported that the serum concentration of miR-21 was significantly increased in patients with COVID-19 compared to healthy controls (85). It was revealed in the present study that the plasma level of miR-21 in patients with COVID-19 was negatively correlated with dendritic cells and senescent memory CD8 T cells suggesting its immunosuppressive properties. Similar immunosuppressive capacity of miR-21 leading to a decrease in cytotoxic T cells was shown in the tumor microenvironment (86).

In a previous study by Wang *et al*, miR-221 was significantly upregulated in the lung tissue of mice with LPS-induced acute lung injury (ALI) . Furthermore, the study revealed that the protective effect of miR-221 on LPS-induced ALI may be mediated by the suppression of the NF-κB pathway (87). Consistent with this, the results of the present study suggest miR-221 as an indicator for lung damage. On days 7 and 14 of follow-up, miR-133a-3p relative expression level was reduced 2-fold and 4-fold, respectively, compared to day 0. The relative level of miR-133a-3p in plasma was positively associated with IL-6, which may indicate myocardial injury. Indeed, the serum level of miR-133 was shown to be positively correlated with IL-6 in patients with the therosclerotic thrombotic cerebral infarction and cardioembolic stroke (88). In the present study, the relative expression of miR-126 was higher than in non-COVID-19 volunteers in contrast to previously published data (85). It was demonstrated that miR-126-3p expression was positively correlated with inflammatory monocyte-derived dendritic cells and negatively with regulatory dendritic cells establishing their pro-inflammatory role in COVID-19. The function of miR-126-5p as a positive regulator of monocyte-mediated inflammatory responses was previously reported (89). In addition, miR-126-3p was the most strongly upregulated in CD14^+^ cells among patients with axial spondyloarthritis (90). The majority of miRNAs have a positive association with the levels of G-CSF and as follows, are involved in inflammation, thus contributing to COVID-19 severity.

In summary, the present study highlights the dynamic changes in different subpopulations of immune cells in patients with severe COVID-19. The collected data indicated the immunodeficiency state and development of the ‘cytokine storm’ syndrome in patients with COVID-19 on day 0 of observation. At the beginning of the observation, lymphopenia and leucopenia were detected in most patients with COVID-19, as well as an increase in the percentage of banded neutrophils, B cells, and CD4^+^ Treg cells, while a decrease in the content of PD-1^low^ T cells, classical, plasmacytoid, and regulatory dendritic cells was also observed. The increased content of different subpopulations of T and dendritic cells starting from the 14th day of hospitalization indicates the activation of the immune response against the progression of inflammation in the lungs. For the first time, dynamic changes in DP CD3^+^CD4^+^CD8^+^ cells, CD127-expressing T cells, CD25-expressing T cells, PD-1^low^ non-T cells, and PD-1^low^ T-cell frequencies have been described in patients with severe COVID-19 pneumonia. The increase of PD-1-positive subpopulations of T and non-T cells and regulatory CD4 T cells in patients with COVID-19 during the observation period suggests the development of an inflammation control mechanism. The positive response after treatment was observed starting from the 7th day, except for T cells, IL-6, TNF-α, and IP-10 levels, which remained increased again from day 14. The high expression levels of miR-21-5p, miR-221-3p, miR-27a-3p, miR-146a-5p, miR-133a-3p, and miR-126-3p at the beginning of hospitalization may contribute to the disease severity. Based on the results of lung CT score correlation analysis, increased concentrations of G-CSF, IP-10, and MIP-1a, as well as the content of lymphocytes, senescent memory T cells, CD127^+^ T cells, and CD57^+^ T cells, had the most prominent impact on post-COVID-19 lung injuries in a long-term period.

## Supplementary Material

Gating strategy for white blood cells, T cells, B cells, natural killer cells, double-positive CD4^+^CD8^+^ T cells, CD3^+^PD-1^low^ T cells, CD3-PD-1^low^ non-T cells, and CD25- and CD127-expressing T cells. PBMCs, peripheral blood mononuclear cells; NK, natural killer; DP, double-positive.

Gating strategy for subpopulations of CD4^+^ and CD8^+^ T cells (memory, effector, activated, senescent, exhausted, regulatory, and senescent memory). PBMCs, peripheral blood mononuclear cells; CTLs, cytotoxic T cells.

Gating strategy for myeloid mononuclear cells subsets: Monocytes, classical DCs, DCs, plasmacytoid DCs, regulatory DCs, inflammatory monocyte-derived DCs. PBMCs, peripheral blood mononuclear cells; DCs, dendritic cells; pDCs, plasmacytoid dendritic cells; Mo-DCs, monocyte-derived dendritic cells.

Correlations between lymphocyte subsets, cytokines, and microRNAs in COVID-19 patients. COVID-19, coronavirus disease 19; CT, computed tomography; ESR, erythrocyte sedimentation rate; IP-10, interferon γ-induced protein 10; MIP-1α, macrophage inflammatory protein-1α; G-CSF, granulocyte colony-stimulating factor; miR, microRNA; WBC, white blood cells; CRP, C-reactive protein; DCs, dendritic cells; NK, natural killer.

Antibodies used for cell characterization by immunofluorescence.

Combinations of markers used for distinct populations of PBMC analysis.

Primer sequences used for miRNA detection.

## Figures and Tables

**Table I:** Clinical characteristics of patients with COVID-19 included in the present study.

Parameters	Patients with COVID-19 (n=14)	non-COVID-19 volunteers (n=17)	P-value
Age, years (median, range)	62 (55-64)	62 (36.2-67)	0.336
Sex			0.200
Male	10/14 (71.42%)	7/17 (41.17%)	
Female	4/14 (28.57%)	10/17 (58.82%)	
The interval from illness onset to hospital admission (days)	11.0 (8.5-12.8)	Not applicable	Not applicable
Underlying diseases, no. (%)			
Hypertension	8/14 (57.14%)	7/17 (41.17%)	0.376
Diabetes	1/14 (7.14%)	2/17 (11.76%)	1.000
Heart disease	6/14 (42.85%)	2/17 (11.76%)	0.097
Symptoms, no. (%)		Not applicable	Not applicable
Fever	10/14 (71.42%)		
Cough	14/14 (100.0%)		
Shortness of breath	11/14 (78.57.0%)		
Diarrhea	1/14 (7.14%)		
Fatigue	14/14 (100.0%)		
Myalgia	3/14 (21.42%)		
Clinical outcome, no. (%)		Not applicable	Not applicable
Recovered and discharged	13/14 (92.85%)		
Death	1/14 (7.14%)		

COVID-19, coronavirus disease 19.

**Table II:** Correlation analysis between computed tomography lesions and certain laboratory parameters.

		Weeks after beginning of hospitalization			
Parameters	Spearman's correlation-coefficient and P-value	2	8	24	48
Lymphocyte count	r	-0.350	-0.461	-0.457	-0.424
	P-value	≤0.05	≤0.005	≤0.005	≤0.005
CD4 T cells	r	-0.372	-0.327	-0.343	-0.360
	P-value	≤0.01	≤0.05	≤0.05	≤0.05
Senescent memory CD4 T cells	r	0.185	0.430	0.382	0.337
	P-value	ns	≤0.005	≤0.05	≤0.05
Senescent memory CD8 T cells	r	0.320	0.500	0.544	0.568
	P-value	≤0.05	≤0.001	≤0.001	≤0.0001
CD3^+^CD127^hi^ cells	r	-0.487	-0.437	-0.453	-0.484
	P-value	≤0.0001	≤0.005	≤0.005	≤0.001
CD3^+^CD57^hi^ cells	r	0.334	0.457	0.469	0.408
	P-value	≤0.05	≤0.005	≤0.001	≤0.01
ESR	r	0.349	0.507	0.507	0.477
	P-value	≤0.05	≤0.001	≤0.001	≤0.001
G-CSF	r	0.308	0.492	0.434	0.397
	P-value	≤0.05	≤0.001	≤0.01	≤0.01
IP-10	r	0.419	0.594	0.599	0.576
	P-value	≤0.005	≤0.0001	≤0.0001	≤0.0001
MIP-1α	r	0.340	0.411	0.414	0.478
	P-value	≤0.05	≤0.01	≤0.005	≤0.001

ESR, erythrocyte sedimentation rate; G-CSF, granulocyte colony-stimulating factor; IP-10, interferon γ-induced protein 10; MIP-1α, macrophage inflammatory protein-1α.

## Data Availability

All datasets presented in this study are provided in the article. Data on correlation analysis may be obtained from the corresponding author upon reasonable request.

## References

[1] Rodriguez-Morales AJ, Cardona-Ospina JA, Gutiérrez-Ocampo E, Villamizar-Peña R, Holguin-Rivera Y, Escalera-Antezana JP, Alvarado-Arnez LE, Bonilla-Aldana DK, Franco-Paredes C, Henao-Martinez AF (2020). Clinical, laboratory and imaging features of COVID-19: A systematic review and meta-analysis. Travel Med Infect Dis.

[2] Ye Q, Wang B, Mao J (2020). The pathogenesis and treatment of the ‘Cytokine Storm’ in COVID-19. J Infect.

[3] Chousterman BG, Swirski FK, Weber GF (2017). Cytokine storm and sepsis disease pathogenesis. Semin Immunopathol.

[4] Islam MT, Nasiruddin M, Khan IN, Mishra SK, Kudrat-E-Zahan M, Riaz TA, Ali ES, Rahman MS, Mubarak MS, Martorell M (2020). A perspective on emerging therapeutic interventions for COVID-19. Front Public Health.

[5] Puneet P, Moochhala S, Bhatia M (2005). Chemokines in acute respiratory distress syndrome. Am J Physiol Lung Cell Mol Physiol.

[6] Bhatia M, Zemans RL, Jeyaseelan S (2012). Role of chemokines in the pathogenesis of acute lung injury. Am J Respir Cell Mol Biol.

[7] Tomashefski JF Jr (2000). Pulmonary pathology of acute respiratory distress syndrome. Clin Chest Med.

[8] Lyu P, Liu X, Zhang R, Shi L, Gao J (2020). The performance of chest CT in evaluating the clinical severity of COVID-19 pneumonia: Identifying critical cases based on CT characteristics. Invest Radiol.

[9] Zhao W, Zhong Z, Xie X, Yu Q, Liu J (2020). Relation between chest CT findings and clinical conditions of coronavirus disease (COVID-19) pneumonia: A multicenter study. AJR Am J Roentgenol.

[10] Wu J, Wu X, Zeng W, Guo D, Fang Z, Chen L, Huang H, Li C (2020). Chest CT findings in patients with coronavirus disease 2019 and its relationship with clinical features. Invest Radiol.

[11] Alon R, Sportiello M, Kozlovski S, Kumar A, Reilly EC, Zarbock A, Garbi N, Topham DJ (2020). Leukocyte trafficking to the lungs and beyond: Lessons from influenza for COVID-19. Nat Rev Immunol.

[12] Zemans RL, Colgan SP, Downey GP (2009). Transepithelial migration of neutrophils: Mechanisms and implications for acute lung injury. Am J Respir Cell Mol Biol.

[13] Zhang L, Yan X, Fan Q, Liu H, Liu X, Liu Z, Zhang Z (2020). D-dimer levels on admission to predict in-hospital mortality in patients with Covid-19. J Thromb Haemost.

[14] Li H, Xiang X, Ren H, Xu L, Zhao L, Chen X, Long H, Wang Q, Wu Q (2020). Serum amyloid A is a biomarker of severe Coronavirus disease and poor prognosis. J Infect.

[15] Gao L, Jiang D, Wen XS, Cheng XC, Sun M, He B, You LN, Lei P, Tan XW, Qin S (2020). Prognostic value of NT-proBNP in patients with severe COVID-19. Respir Res.

[16] Al Balushi A, Al Shekaili J, Al Kindi M, Ansari Z, Al-Khabori M, Khamis F, Ambusaidi Z, Al Balushi A, Al Huraizi A, Al Sulaimi S (2021). Immunological predictors of disease severity in patients with COVID-19. Int J Inf Dis.

[17] Sidiropoulou P, Docea AO, Nikolaou V, Katsarou MS, Spandidos DA, Tsatsakis A, Calina D, Drakoulis N (2021). Unraveling the roles of vitamin D status and melanin during COVID-19 (Review). Int J Mol Med.

[18] Hussain T, Zhao D, Shah SZA, Wang J, Yue R, Liao Y, Sabir N, Yang L, Zhou X (2018). MicroRNA 27a-3p regulates antimicrobial responses of murine macrophages infected by mycobacterium avium subspecies paratuberculosis by targeting interleukin-10 and TGF-β-activated protein kinase 1 binding protein 2. Front Immunol.

[19] de Gonzalo-Calvo D, Benitez ID, Pinilla L, Carratala A, Moncusi-Moix ANNA, Gort-Paniello C, Molinero M, González J, Torres G, Bernal M (2021). Circulating microRNA profiles predict the severity of COVID-19 in hospitalized patients. Transl Res.

[20] Lev S, Gottesman T, Levin GS, Lederfein D, Berkov E, Diker D, Zaidman A, Nutman A, Ber AI, Angel A (2021). Observational cohort study of IP-10's potential as a biomarker to aid in inflammation regulation within a clinical decision support protocol for patients with severe COVID-19. PLoS One.

[21] Iannetta M, Buccisano F, Fraboni D, Malagnino V, Campogiani L, Teti E, Spalliera I, Rossi B, Di Lorenzo A, Palmieri R (2021). Baseline T-lymphocyte subset absolute counts can predict both outcome and severity in SARS-CoV-2 infected patients: A single center study. Sci Rep.

[22] Deng R, Wang C, Ye Y, Gou L, Fu Z, Ye B, Shao F, Zhang X, Fu W, Xiao J (2021). Clinical manifestations of blood cell parameters and inflammatory factors in 92 patients with COVID-19. Ann Transl Med.

[23] Shi H, Wang W, Yin J, Ouyang Y, Pang L, Feng Y, Qiao L, Guo X, Shi H, Jin R, Chen D (2020). The inhibition of IL-2/IL-2R gives rise to CD8 + T cell and lymphocyte decrease through JAK1-STAT5 in critical patients with COVID-19 pneumonia. Cell Death Dis.

[24] Liao M, Liu Y, Yuan J, Wen Y, Xu G, Zhao J, Cheng L, Li J, Wang X, Wang F (2020). Single-cell landscape of bronchoalveolar immune cells in patients with COVID-19. Nat Med.

[25] https://www.who.int/publications/i/item/10665-332299.

[26] Livak KJ, Schmittgen TD (2001). Analysis of relative gene expression data using real-time quantitative PCR and the 2(-Delta Delta C(T)) method. Methods.

[27] Büttner L, Aigner A, Fleckenstein FN, Hamper CM, Jonczyk M, Hamm B, Scholz O, Böning G (2020). Diagnostic value of initial chest CT findings for the need of ICU treatment/intubation in patients with COVID-19. Diagnostics (Basel).

[28] Liu J, Li S, Liu J, Liang B, Wang X, Wang H, Li W, Tong Q, Yi J, Zhao L (2020). Longitudinal characteristics of lymphocyte responses and cytokine profiles in the peripheral blood of SARS-CoV-2 infected patients. EBioMedicine.

[29] Huang C, Wang Y, Li X, Ren L, Zhao J, Hu Y, Zhang L, Fan G, Xu J, Gu X (2020). Clinical features of patients infected with 2019 novel coronavirus in Wuhan, China. Lancet.

[30] Lax SF, Skok K, Zechner P, Kessler HH, Kaufmann N, Koelblinger C, Vander K, Bargfrieder U, Trauner M (2020). Pulmonary arterial thrombosis in COVID-19 with fatal outcome: Results from a prospective, single-center, clinicopathologic case series. Ann Intern Med.

[31] Xu Z, Shi L, Wang Y, Zhang J, Huang L, Zhang C, Liu S, Zhao P, Liu H, Zhu L (2020). Pathological findings of COVID-19 associated with acute respiratory distress syndrome. Lancet Respir Med.

[32] Marté JL, Toney NJ, Cordes L, Schlom J, Donahue RN, Gulley JL (2020). Original research: Early changes in immune cell subsets with corticosteroids in patients with solid tumors: Implications for COVID-19 management. J Immunother Cancer.

[33] Abdin SM, Elgendy SM, Alyammahi SK, Alhamad DW, Omar HA (2020). Tackling the cytokine storm in COVID-19, challenges and hopes. Life Sci.

[34] Tomić S, Đokić J, Stevanović D, Ilić N, Gruden-Movsesijan A, Dinić M, Radojević D, Bekić M, Mitrović N, Tomašević R (2021). Reduced expression of autophagy markers and expansion of myeloid-derived suppressor cells correlate with poor T cell response in severe COVID-19 patients. Front Immunol.

[35] Deng Z, Zhang M, Zhu T, Zhili N, Liu Z, Xiang R, Zhang W, Xu Y (2020). Dynamic changes in peripheral blood lymphocyte subsets in adult patients with COVID-19. Int J Infect Dis.

[36] Riley JL (2009). PD-1 signaling in primary T cells. Immunol Rev.

[37] Pyzik A, Grywalska E, Matyjaszek-Matuszek B, Smoleń A, Pyzik D, Roliński J (2017). Frequencies of PD-1-positive T CD3+CD4+, T CD3+CD8+ and B CD19+ lymphocytes in female patients with Graves' disease and healthy controls-preliminary study. Mol Cell Endocrinol.

[38] Nascimbeni M, Shin EC, Chiriboga L, Kleiner DE, Rehermann B (2004). Peripheral CD4(+)CD8(+) T cells are differentiated effector memory cells with antiviral functions. Blood.

[39] Overgaard NH, Jung JW, Steptoe RJ, Wells JW (2015). CD4+/CD8+ double-positive T cells: More than just a developmental stage?. J Leukoc Biol.

[40] Yang X, Dai T, Zhou X, Qian H, Guo R, Lei L, Zhang X, Zhang D, Shi L, Cheng Y (2020). Naturally activated adaptive immunity in COVID-19 patients. J Cell Mol Med.

[41] Kalfaoglu B, Almeida-Santos J, Tye CA, Satou Y, Ono M (2020). T-cell hyperactivation and paralysis in severe COVID-19 infection revealed by single-cell analysis. Front Immunol.

[42] Chen X, Huang J, Huang Y, Chen J, Huang Y, Jiang X, Shi Y (2020). Characteristics of immune cells and cytokines in patients with coronavirus disease 2019 in Guangzhou, China. Hum Immunol.

[43] Neidleman J, Luo X, Frouard J, Xie G, Gill G, Stein ES, McGregor M, Ma T, George AF, Kosters A (2020). SARS-CoV-2-Specific T cells exhibit phenotypic features of helper function, lack of terminal differentiation, and high proliferation potential. Cell Rep Med.

[44] Wang W, Xiang HP, Wang HP, Zhu LX, Geng XP (2017). CD4 + CD25 + CD127 high cells as a negative predictor of multiple organ failure in acute pancreatitis. World J Emerg Surg.

[45] Kalfaoglu B, Almeida-Santos J, Tye CA, Satou Y, Ono M (2021). T-cell dysregulation in COVID-19. Biochem Biophys Res Commun.

[46] Hanna SJ, Codd AS, Gea-Mallorqui E, Scourfield DO, Richter FC, Ladell K, Borsa M, Compeer EB, Moon OR, Galloway SAE (2021). T cell phenotypes in COVID-19-a living review. Oxf Open Immunol.

[47] Kusnadi A, Ramírez-Suástegui C, Fajardo V, Chee SJ, Meckiff BJ, Simon H, Pelosi E, Seumois G, Ay F, Vijayanand P, Ottensmeier CH (2021). Severely ill COVID-19 patients display impaired exhaustion features in SARS-CoV-2-reactive CD8+ T cells. Sci Immunol.

[48] Peng Y, Mentzer AJ, Liu G, Yao X, Yin Z, Dong D, Dejnirattisai W, Rostron T, Supasa P, Liu C (2020). Broad and strong memory CD4+ and CD8+ T cells induced by SARS-CoV-2 in UK convalescent individuals following COVID-19. Nat Immunol.

[49] Neagu M, Calina D, Docea AO, Constantin C, Filippini T, Vinceti M, Drakoulis N, Poulas K, Nikolouzakis TK, Spandidos DA, Tsatsakis A (2021). Back to basics in COVID-19: Antigens and antibodies-completing the puzzle. J Cell Mol Med.

[50] De Biasi S, Meschiari M, Gibellini L, Bellinazzi C, Borella R, Fidanza L, Gozzi L, Iannone A, Lo Tartaro D, Mattioli M (2020). Marked T cell activation, senescence, exhaustion and skewing towards TH17 in patients with COVID-19 pneumonia. Nat Commun.

[51] Qin C, Zhou L, Hu Z, Zhang S, Yang S, Tao Y, Xie C, Ma K, Shang K, Wang W, Tian DS (2020). Dysregulation of immune response in patients with COVID-19 in Wuhan, China. Clin Infect Dis.

[52] Wang F, Hou H, Luo Y, Tang G, Wu S, Huang M, Liu W, Zhu Y, Lin Q, Mao L (2020). The laboratory tests and host immunity of COVID-19 patients with different severity of illness. JCI Insight.

[53] Chen G, Wu D, Guo W, Cao Y, Huang D, Wang H, Wang T, Zhang X, Chen H, Yu H (2020). Clinical and immunological features of severe and moderate coronavirus disease 2019. J Clin Invest.

[54] Yang J, Zhang E, Zhong M, Yang Q, Hong K, Shu T, Zhou D, Xiang J, Xia J, Zhou X (2020). Longitudinal characteristics of t cell responses in asymptomatic SARS-CoV-2 infection. Virol Sin.

[55] Sanchez-Cerrillo I, Landete P, Aldave B, Sanchez-Alonso S, Sanchez-Azofra A, Marcos-Jimenez A, Avalos E, Alcaraz-Serna A, de Los Santos I, Mateu-Albero T (2020). Differential redistribution of activated monocyte and dendritic cell subsets to the lung associates with severity of COVID-19. medRxiv.

[56] Leng Z, Zhu R, Hou W, Feng Y, Yang Y, Han Q, Shan G, Meng F, Du D, Wang S (2020). Transplantation of ACE2- mesenchymal stem cells improves the outcome of patients with COVID-19 pneumonia. Aging Dis.

[57] Fujita S, Seino KI, Sato K, Sato Y, Eizumi K, Yamashita N, Taniguchi M, Sato K (2006). Regulatory dendritic cells act as regulators of acute lethal systemic inflammatory response. Blood.

[58] Boor PPC, Metselaar HJ, Mancham S, Tilanus HW, Kusters JG, Kwekkeboom J (2006). Prednisolone suppresses the function and promotes apoptosis of plasmacytoid dendritic cells. Am J Transplant.

[59] Shin KS, Jeon I, Kim BS, Kim IK, Park YJ, Koh CH, Song B, Lee JM, Lim J, Bae EA (2019). Monocyte-derived dendritic cells dictate the memory differentiation of CD8+ T cells during acute infection. Front Immunol.

[60] Calina D, Sarkar C, Arsene AL, Salehi B, Docea AO, Mondal M, Islam MT, Zali A, Sharifi-Rad J (2020). Recent advances, approaches and challenges in targeting pathways for potential COVID-19 vaccines development. Immunol Res.

[61] Aldridge JR Jr, Moseley CE, Boltz DA, Negovetich NJ, Reynolds C, Franks J, Brown SA, Doherty PC, Webster RG, Thomas PG (2009). From the cover: TNF/iNOS-producing dendritic cells are the necessary evil of lethal influenza virus infection. Proc Natl Acad Sci USA.

[62] Iijima N, Mattei LM, Iwasaki A (2011). Recruited inflammatory monocytes stimulate antiviral Th1 immunity in infected tissue. Proc Natl Acad Sci USA.

[63] Haroun RAH, Osman WH, Eessa AM (2021). Interferon-γ-induced protein 10 (IP-10) and serum amyloid A (SAA) are excellent biomarkers for the prediction of COVID-19 progression and severity. Life Sci.

[64] Wang G, Wu C, Zhang Q, Wu F, Yu B, Lv J, Li Y, Li T, Zhang S, Wu C (2020). C-reactive protein level may predict the risk of COVID-19 aggravation. Open Forum Infect Dis.

[65] Chen W, Zheng KI, Liu S, Yan Z, Xu C, Qiao Z (2020). Plasma CRP level is positively associated with the severity of COVID-19. Ann Clin Microbiol Antimicrob.

[66] Mardani R, Namavar M, Ghorbi E, Shoja Z, Zali F, Kaghazian H, Aghasadeghi MR, Sadeghi SA, Sabeti S, Darazam IA (2022). Association between serum inflammatory parameters and the disease severity in COVID-19 patients. J Clin Lab Anal.

[67] Peruzzi B, Bencini S, Capone M, Mazzoni A, Maggi L, Salvati L, Vanni A, Orazzini C, Nozzoli C, Morettini A (2020). Quantitative and qualitative alterations of circulating myeloid cells and plasmacytoid DC in SARS-CoV-2 infection. Immunology.

[68] Mangano C, Oliva BM (2021). Relationship between lymphocyte subsets values and C-reactive protein in COVID-19 patients. Cytometry A.

[69] Tyurin AV, Salimgareeva MK, Miniakhmetov IR, Khusainova RI, Samorodov A, Pavlov VN, Kzhyshkowska J (2022). Correlation of the imbalance in the circulating lymphocyte subsets with c-reactive protein and cardio-metabolic conditions in patients with COVID-19. Front Immunol.

[70] Meizlish ML, Pine AB, Bishai JD, Goshua G, Nadelmann ER, Simonov M, Chang CH, Zhang H, Shallow M, Bahel P (2021). A neutrophil activation signature predicts critical illness and mortality in COVID-19. Blood Adv.

[71] Hasanvand A (2022). COVID-19 and the role of cytokines in this disease. Inflammopharmacology.

[72] Tavakkoli M, Wilkins CR, Mones JV, Mauro MJ (2019). A novel paradigm between leukocytosis, G-CSF secretion, neutrophil-to-lymphocyte ratio, myeloid-derived suppressor cells, and prognosis in non-small cell lung cancer. Front Oncol.

[73] Miles B

[74] Chen Y, Wang J, Liu C, Su L, Zhang D, Fan J, Yang Y, Xiao M, Xie J, Xu Y (2020). IP-10 and MCP-1 as biomarkers associated with disease severity of COVID-19. Mol Med.

[75] Pons MJ, Ymaña B, Mayanga-Herrera A, Sáenz Y, Alvarez-Erviti L, Tapia-Rojas S, Gamarra R, Blanco AB, Moncunill G, Ugarte-Gil MF (2021). Cytokine profiles associated with worse prognosis in a hospitalized peruvian COVID-19 cohort. Front Immunol.

[76] Tjan LH, Furukawa K, Nagano T, Kiriu T, Nishimura M, Arii J, Hino Y, Iwata S, Nishimura Y, Mori Y (2021). Early differences in cytokine production by severity of coronavirus disease 2019. J Infect Dis.

[77] Yang AP, Li HM, Tao WQ, Yang XJ, Wang M, Yang WJ, Liu JP (2020). Infection with SARS-CoV-2 causes abnormal laboratory results of multiple organs in patients. Aging (Albany NY).

[78] Liu Y, Tan W, Chen H, Zhu Y, Wan L, Jiang K, Guo Y, Tang K, Xie C, Yi H (2021). Dynamic changes in lymphocyte subsets and parallel cytokine levels in patients with severe and critical COVID-19. BMC Infect Dis.

[79] Leisman DE, Ronner L, Pinotti R, Taylor MD, Sinha P, Calfee CS, Hirayama AV, Mastroiani F, Turtle CJ, Harhay MO (2020). Cytokine elevation in severe and critical COVID-19: A rapid systematic review, meta-analysis, and comparison with other inflammatory syndromes. Lancet Respir Med.

[80] Han H, Ma Q, Li C, Liu R, Zhao L, Wang W, Zhang P, Liu X, Gao G, Liu F (2020). Profiling serum cytokines in COVID-19 patients reveals IL-6 and IL-10 are disease severity predictors. Emerg Microbes Infect.

[81] Lambert KA, Roff AN, Panganiban RP, Douglas S, Ishmael FT (2018). MicroRNA-146a is induced by inflammatory stimuli in airway epithelial cells and augments the anti-inflammatory effects of glucocorticoids. PLoS One.

[82] Taganov KD, Boldin MP, Chang KJ, Baltimore D (2006). NF-κB-dependent induction of microRNA miR-146, an inhibitor targeted to signaling proteins of innate immune responses. Proc Natl Acad Sci USA.

[83] Zeng Z, Gong H, Li Y, Jie K, Ding C, Shao Q, Liu F, Zhan Y, Nie C, Zhu W, Qian K (2013). Upregulation of miR-146a contributes to the suppression of inflammatory responses in LPS-induced acute lung injury. Exp Lung Res.

[84] Donyavi T, Bokharaei-Salim F, Baghi HB, Khanaliha K, Janat-Makan MA, Karimi B, Nahand JS, Mirzaei H, Khatami A, Garshasbi S (2021). Acute and post-acute phase of COVID-19: Analyzing expression patterns of miRNA-29a-3p, 146a-3p, 155-5p, and let-7b-3p in PBMC. Int Immunopharmacol.

[85] Garg A, Seeliger B, Derda AA, Xiao K, Gietz A, Scherf K, Sonnenschein K, Pink I, Hoeper MM, Welte T (2021). Circulating cardiovascular microRNAs in critically ill COVID-19 patients. Eur J Heart Fail.

[86] Meng G, Wei J, Wang Y, Qu D, Zhang J (2020). MiR-21 regulates immunosuppression mediated by myeloid-derived suppressor cells by impairing RUNX1-YAP interaction in lung cancer. Cancer Cell Int.

[87] Wang T, Jiang L, Wei X, Dong Z, Liu B, Zhao J, Wang L, Xie P, Wang Y, Zhou S (2019). Inhibition of miR-221 alleviates LPS-induced acute lung injury via inactivation of SOCS1/NF-κB signaling pathway. Cell Cycle.

[88] Xu P, Xin J, Song L, Chen Y, Ma J, Liu L, Qi Z, Pan X, Zhou S (2020). Serum miR-133 as a potential biomarker in acute cerebral infarction patients. Clin Lab.

[89] Huang J, Zhu L, Qiu C, Xu X, Zhang L, Ding X, Liao Q, Xu J, Zhang X (2017). MicroRNA miR-126-5p enhances the inflammatory responses of monocytes to lipopolysaccharide stimulation by suppressing cylindromatosis in chronic HIV-1 Infection. J Virol.

[90] Fogel O, Tinggaard AB, Fagny M, Sigrist N, Roche E, Leclere L, Deleuze JF, Batteux F, Dougados M, Miceli-Richard C, Tost J (2019). Deregulation of microRNA expression in monocytes and CD4+ T lymphocytes from patients with axial spondyloarthritis. Arthritis Res Ther.

