# Supplementary material for: Dynamic changes in radiological parameters, immune cells, selected miRNAs, and cytokine levels in peripheral blood of patients with severe COVID‑19
Source: Biomed Rep. 2023 Mar 21;18(5):33. doi: 10.3892/br.2023.1615 (PMC10074022; doi:10.3892/br.2023.1615)

Figure S1. Gating strategy for white blood cells, T cells, B cells, natural killer cells, double-positive CD4⁺CD8⁺ T cells, CD3⁺PD-1^{low} T cells, CD3-PD-1^{low} non-T cells, and CD25- and CD127-expressing T cells. PBMCs, peripheral blood mononuclear cells; NK, natural killer; DP, double-positive.

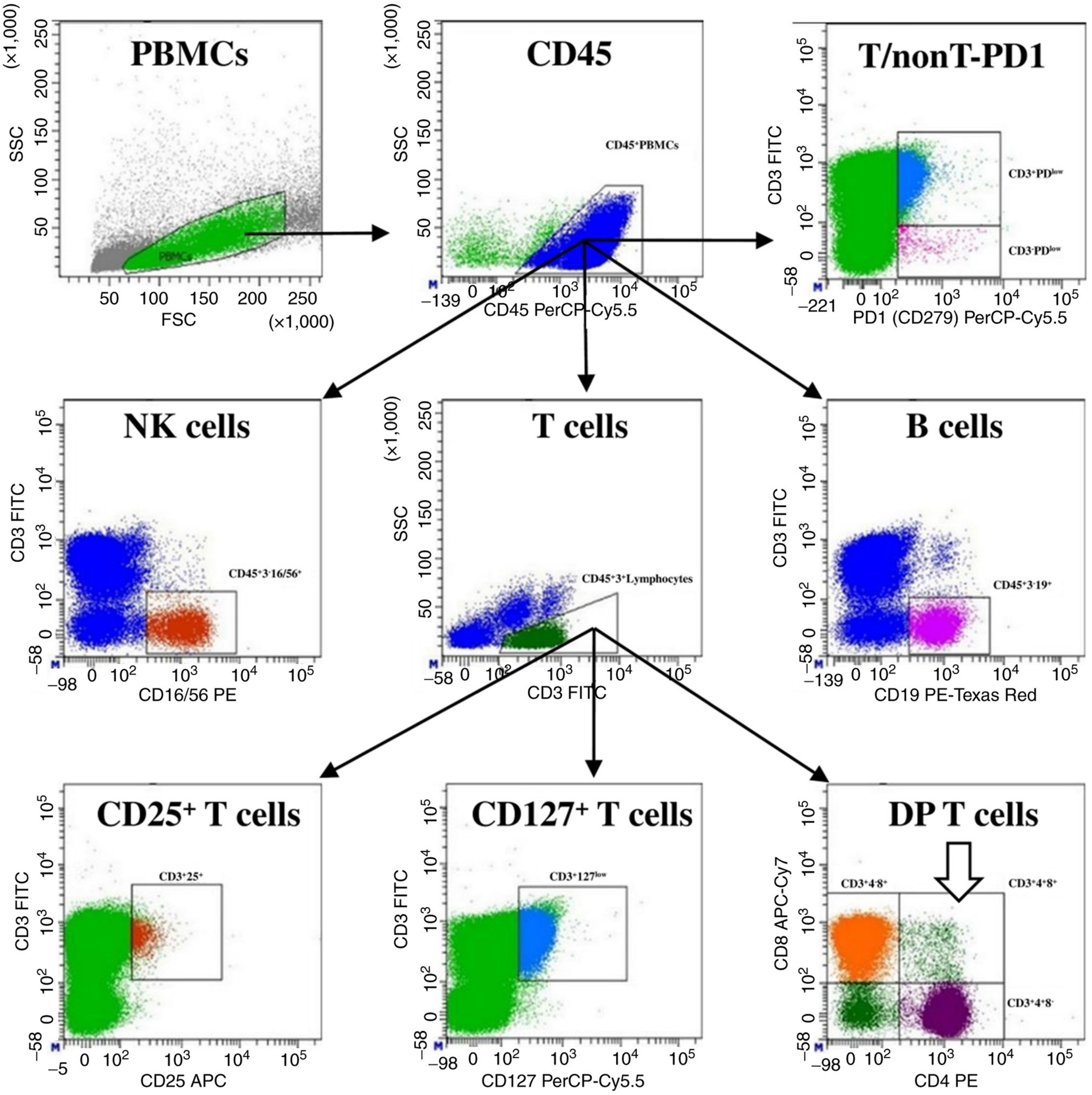

Figure S2. Gating strategy for subpopulations of CD4⁺ and CD8⁺ T cells (memory, effector, activated, senescent, exhausted, regulatory, and senescent memory). PBMCs, peripheral blood mononuclear cells; CTLs, cytotoxic T cells.

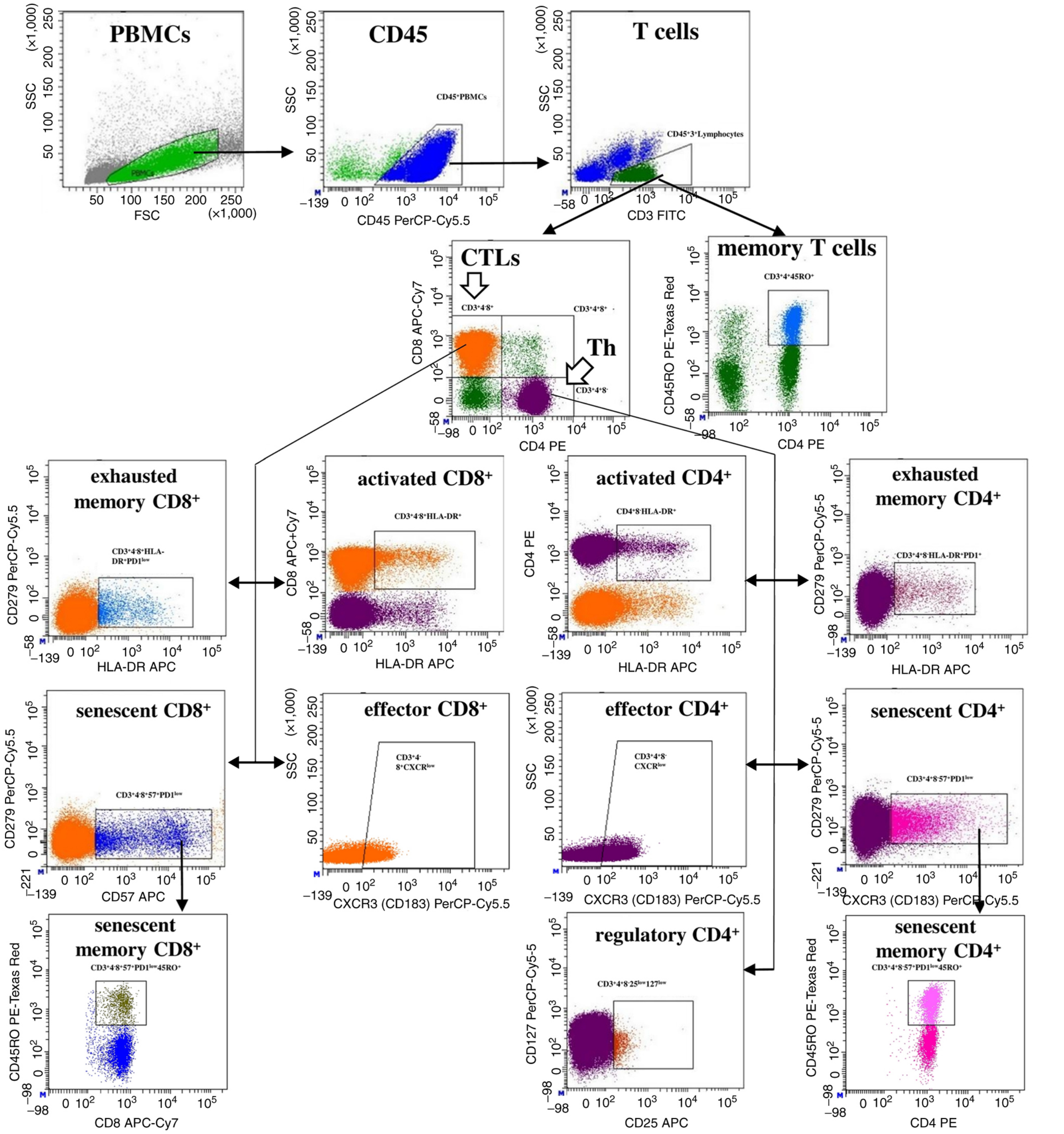

Figure S3. Gating strategy for myeloid mononuclear cells subsets: Monocytes, classical DCs, DCs, plasmacytoid DCs, regulatory DCs, inflammatory monocyte-derived DCs. PBMCs, peripheral blood mononuclear cells; DCs, dendritic cells; pDCs, plasmacytoid dendritic cells; Mo-DCs, monocyte-derived dendritic cells.

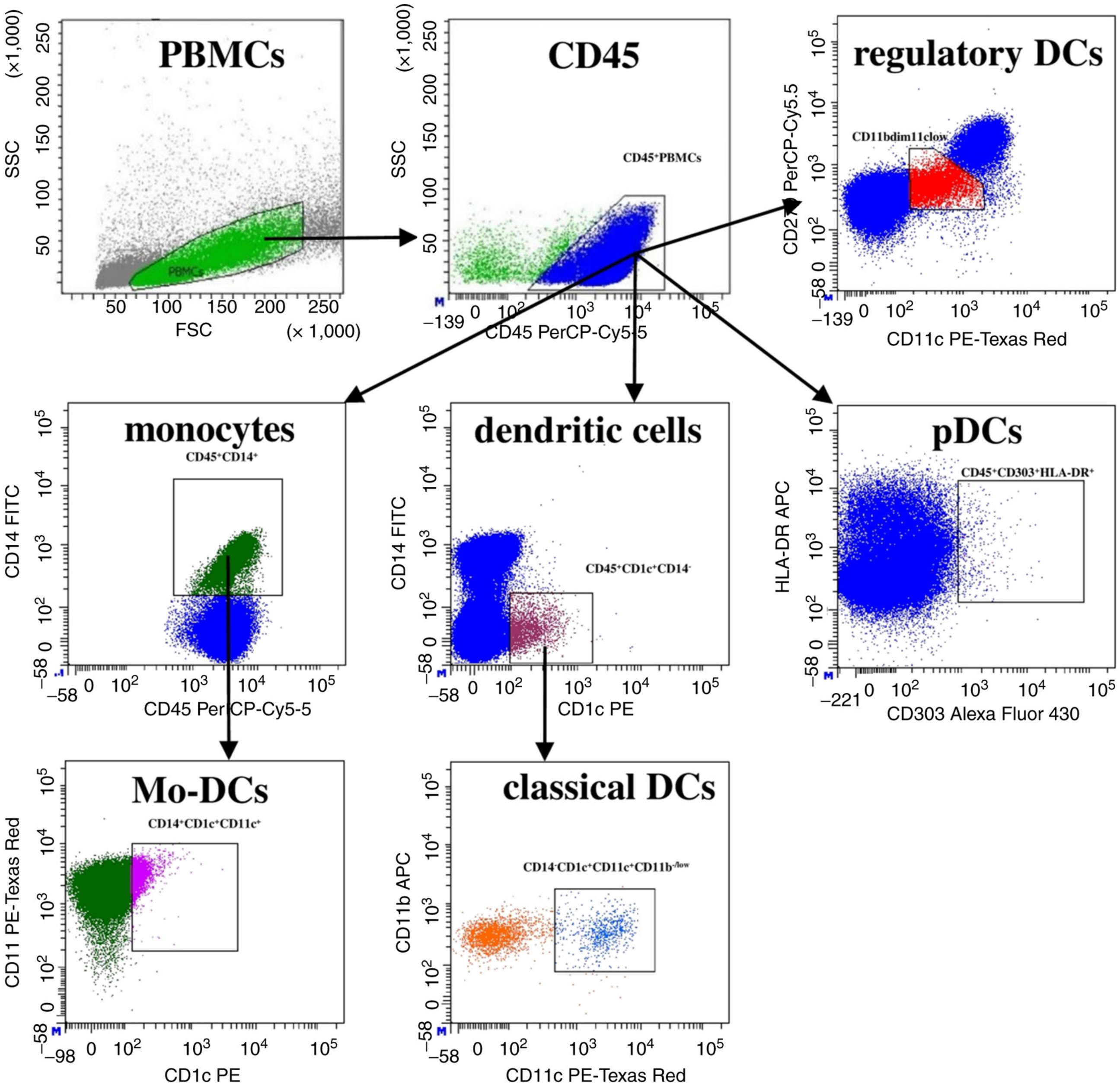

Figure S4. Correlations between lymphocyte subsets, cytokines, and microRNAs in COVID-19 patients. COVID-19, coronavirus disease 19; CT, computed tomography; ESR, erythrocyte sedimentation rate; IP-10, interferon γ-induced protein 10; MIP-1α, macrophage inflammatory protein-1α; G-CSF, granulocyte colony-stimulating factor; miR, microRNA; WBC, white blood cells; CRP, C-reactive protein; DCs, dendritic cells; NK, natural killer.

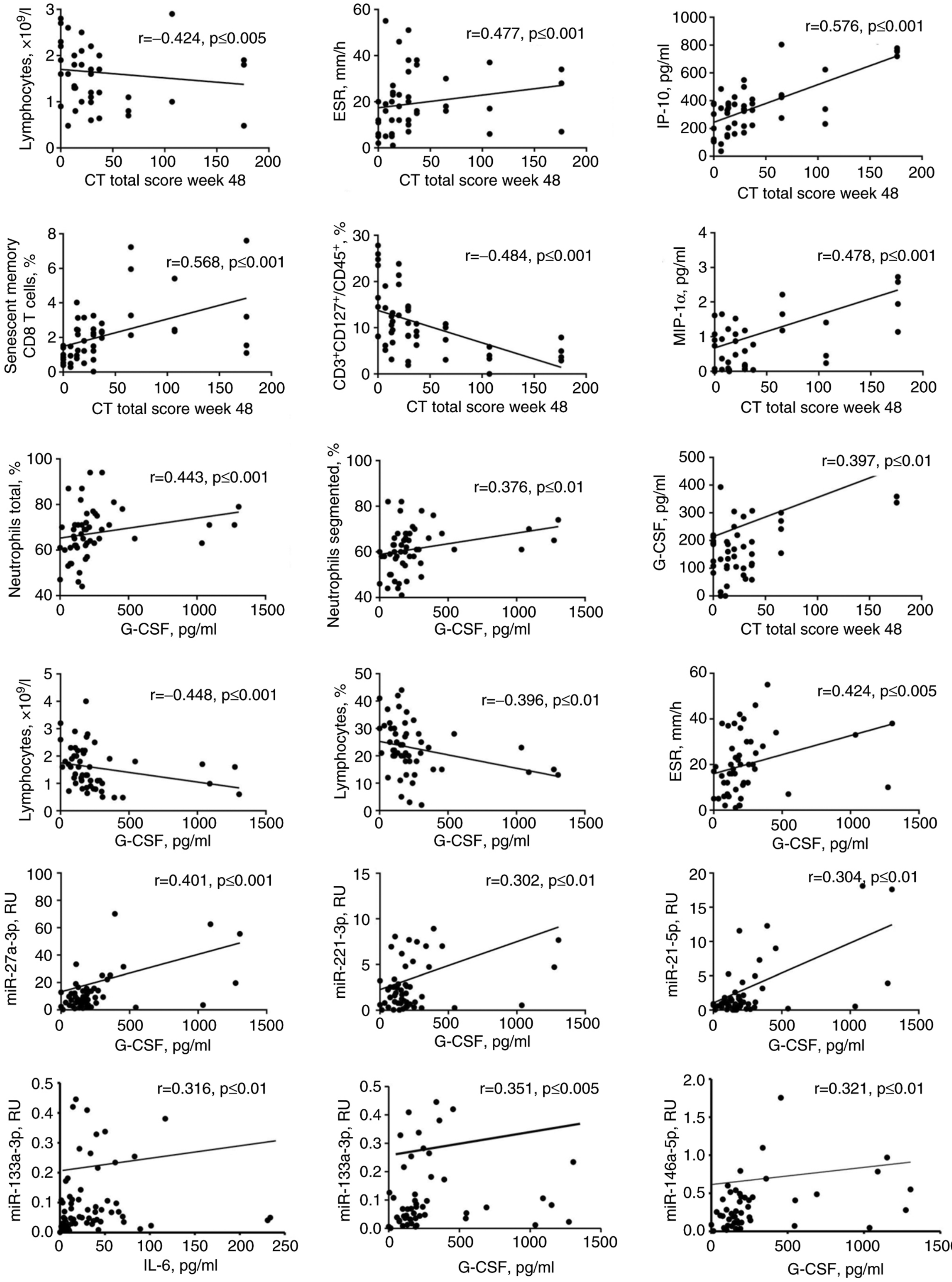

Figure S4. Continued.

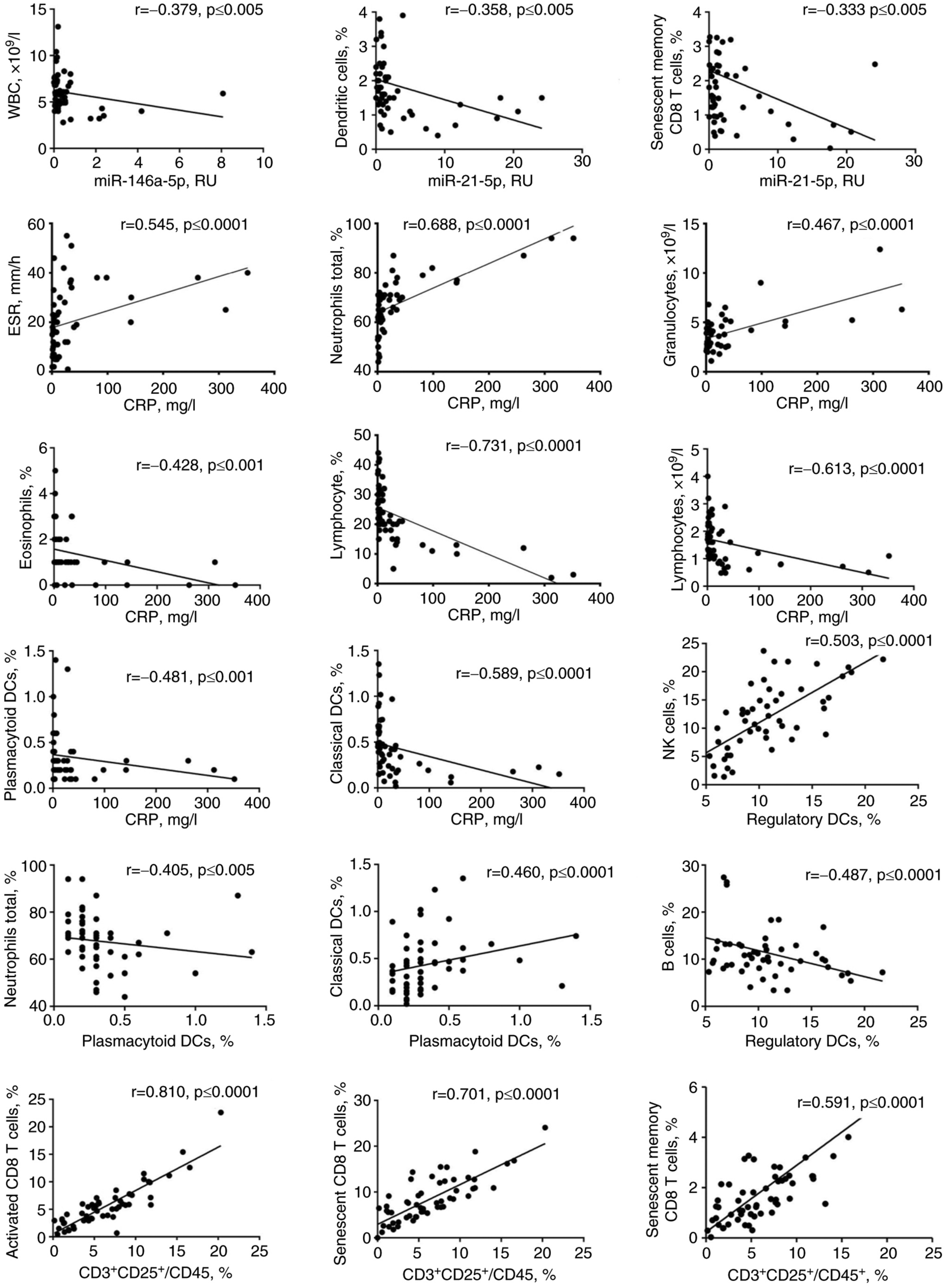

Figure S4. Continued.

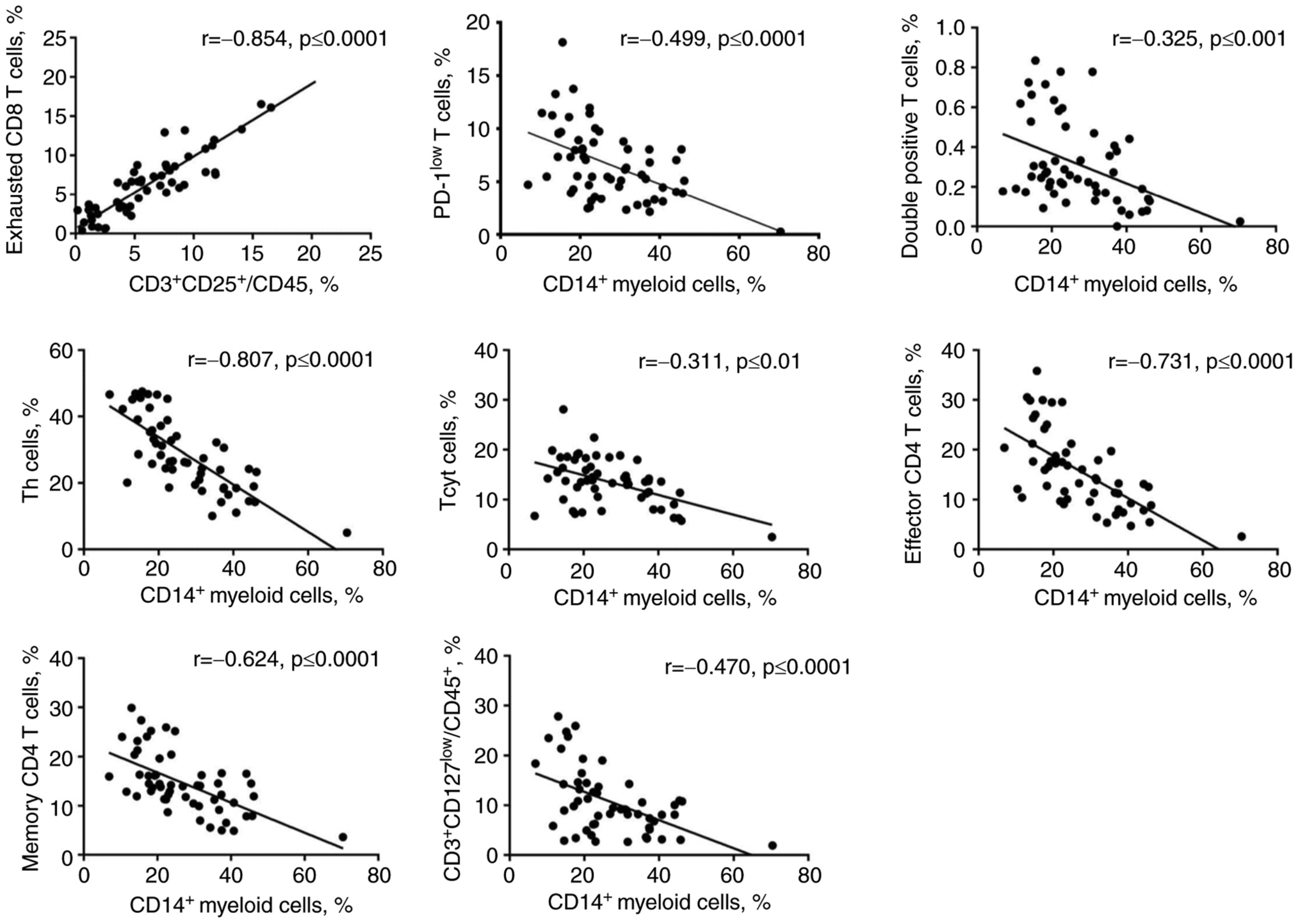

Supplement: Gating strategy for white blood cells, T cells, B cells, natural killer cells, double-positive CD4+CD8+ T cells, CD3+PD-1low T cells, CD3-PD-1low non-T cells, and CD25- and CD127-expressing T cells. PBMCs, peripheral blood mononuclear cells; NK, natural killer; DP, double-positive. [file Supplementary_Data1.pdf]
