# Supplementary material for: Dynamic changes in radiological parameters, immune cells, selected miRNAs, and cytokine levels in peripheral blood of patients with severe COVID‑19
Source: Biomed Rep. 2023 Mar 21;18(5):33. doi: 10.3892/br.2023.1615 (PMC10074022; doi:10.3892/br.2023.1615)
Supplement: Antibodies used for cell characterization by immunofluorescence. [file Supplementary_Data2.pdf]

Table SI. Antibodies used for cell characterization by immunofluorescence.

CD	Fluorophore	Clone	Cat. no.	Lot	Cell subset
CD1c	PE	F10/21A3	564900	0162405	Myeloid DCs
CD3	FITC	HIT3a	555339	0064351	T cells
CD4	PE	RPA-T4	555347	0128475	T helper
CD8	APC-Cy7	SK1	557834	0247872	CTLs (T-cell subsets)
CD11b	APC	ICRF44	550019	0128389	DCs
CD11c	PE-CF594	B-ly6	562393	0108833	DCs
CD14	FITC	M5E2	555397	9315119	Monocytes
CD16	PE	B73.1	561313	0073839	NK
CD19	PE-CF594	HIB19	562294	0230909	B cells
CD25	APC	M-A251	555434	0064948	Regulatory T cells
CD45	PerCP-Cy™5.5	HI30	564105	0216944	PBMCs
CD45RO	PE-CF594	UCHL1	562299	0293276	T memory
CD56	PE	B159	555516	0160477	NK
CD57	APC	NK-1	560845	0212460	Senescent cells
CD127 (IL-7Rα)	PerCP-Cy™5.5	HIL-7R-M21	560551	0142744	Memory and effector T cells
CD183 (CXCR3)	PerCP-Cy™5.5	1C6/CXCR3	560832	0279904	T effector
CD279 (PD-1)	PerCP-Cy™5.5	EH12.1	561273	0300813	Senescent cells
CD303 (BDCA-2)	BV510	V24-785	748005	0323865	Plasmacytoid DCs
HLA-DR	APC	G46-6	559866	0028348	Activated cells

All of the antibodies were obtained from BD Biosciences. CD, cluster of differentiation; DCs, dendritic cells; CTLs, cytotoxic T lymphocytes; NK, natural killer; PBMCs, peripheral blood mononuclear cells.

Table SII. Combinations of markers used for distinct populations of PBMC analysis.

Gating	Cell subset
$CD45^+$/SSC	$CD45^+$ PBMCs
$CD45^+CD14^+$	Monocytes
$CD45^+CD14^-CD1c^+$	DCs
$CD45^+CD14^+D11c^+CD1c^{low}$	Inflammatory monocyte-derived DCs
$CD45^+CD14^-CD1c^+CD11c^+CD11b^{low}$	Classical DCs
$CD45^+CD14^+CD11c^{low}CD11b^{dim}$	Regulatory DCs
$CD45^+CD303^+HLA\text{-}DR^+$	Plasmacytoid DCs
$CD45^+CD3^+$/SSC	T cells
$CD45^+CD3^-CD19^+$	B cells
$CD45^+CD3^-CD16^+CD56^+$	NK cells
$CD3^+CD4^+CD8^-$	Th cells
$CD3^+CD4^+$CD45RO	Memory T cells
$CD3^+CD4^+CD8^-CXCR3^{low}$	Effector $CD4^+$ T cells
$CD3^+CD4^+CD8^-HLA\text{-}DR^+$	Activated $CD4^+$ T cells
$CD3^+CD4^+CD8^-CD57^+PD\text{-}1^{low}$	Senescent $CD4^+$ T cells
$CD3^+CD4^+CD8^-HLA\text{-}DR^+$ $PD\text{-}1^{low}$	Exhausted $CD4^+$ T cells
$CD3^+CD4^+CD8^-CD57^+PD\text{-}1^{low}CD45RO^+$	Senescent memory $CD4^+$ T cells
$CD3^+CD4^+CD8^-CD25^{low}CD127^{low}$	Regulatory $CD4^+$ T cells
$CD3^+CD4^-CD8^+$	Cytotoxic T lymphocytes
$CD3^+CD4^-CD8^+CXCR3^{low}$	Effector $CD8^+$ T cells
$CD3^+CD4^-CD8^+HLA\text{-}DR^+$	Activated $CD8^+$ T cells
$CD3^+CD4^-CD8^+CD57^+PD\text{-}1^{-/low}$	Senescent $CD8^+$ T cells
$CD3^+CD4^-CD8^+CD57^+PD\text{-}1^{-/low}CD45RO^+$	Senescent memory $CD8^+$ T cells
$CD3^+CD4^-CD8^+HLA\text{-}DR^+$ $PD\text{-}1^{-/low}$	Exhausted $CD8^+$ T cells
$CD3^+CD4^+CD8^+$	Double-positive T-cells
$CD3^+PD\text{-}1^{low}$	$PD1^{low}$ T cells
$CD3^-PD\text{-}1^{low}$	$PD1^{low}$ non-T cells
$CD3^+CD25^+$	$CD25^+$ (IL2R) T cells
$CD3^+CD127^{low}$	$CD127^{low}$ (IL7R) T cells

PBMC, peripheral blood mononuclear cell; DCs, dendritic cells.; NK, natural killer.

Table SIII. Primer sequences used for miRNA detection.

Name	Forward	Reverse
hsa-miR-21-5p	5'-TAGCTTATCAGACTGATGTTGA-3'	Universal reverse primer kit[a]
hsa-miR-27a-3p	5'-TTCACAGTGGCTAAGTTCCGC-3'	
hsa-miR-126-3p	5'-TCGTACCGTGAGTAATAATGCG-3'	
hsa-miR-133a-3p	5'-TTTGGTCCCCTTCAACCAGCTG-3'	
hsa-miR-146a-5p	5'-TGAGAACTGAATTCCATGGGTT-3'	
hsa-miR-221-3p	5'-AGCTACATTGTCTGCTGGGTTTC-3'	
U6	5'-CTCGCTTCGGCAGCACA-3'	5'-AACGCTTCACGAATTTGCGT-3'

[a]miRNA 1st Strand cDNA Synthesis kit; cat. no. 600036; Agilent Technologies, Inc. miRNA or miR, microRNA.
